# *Trans*-Membrane Area Asymmetry Controls the Shape of Cellular Organelles

**DOI:** 10.3390/ijms16035299

**Published:** 2015-03-09

**Authors:** Galina V. Beznoussenko, Sergei S. Pilyugin, Willie J. C. Geerts, Michael M. Kozlov, Koert N. J. Burger, Alberto Luini, Jure Derganc, Alexander A. Mironov

**Affiliations:** 1 The FIRC Institute of Molecular Oncology, Milan 20139, Italy; E-Mail: galina.beznusenko@ifom.eu; 2 Consorzio Mario Negri Sud, S. Maria Imbaro, Chieti 66030, Italy; 3 Department of Mathematics, University of Florida, Gainesville, FL 32611-8105, USA; E-Mail: pilyugin@math.ufl.edu; 4 Department of Biochemical Physiology, Institute of Biomembranes, 3584 CH Utrecht, The Netherlands; E-Mails: w.geerts@bio.uu.nl (W.J.C.G.); koert.burger@hccnet.nl (K.N.J.B.); 5 Department of Physiology and Pharmacology, Tel Aviv University, Tel Aviv 69978, Israel; E-Mail: misha@picard.tau.ac.il; 6 Consiglio Nazionale delle Ricerche (CNR), Istituto di Biochimica delle Proteine, Naples 80131, Italy; E-Mail: luini@tigem.it; 7 Institute of Biophysics, University of Ljubljana, 1000 Ljubljana, Slovenia

**Keywords:** intra-Golgi transport, Golgi apparatus, lipid membrane, *trans*-membrane area asymmetry, organelle shape, kiss-and-run model

## Abstract

Membrane organelles often have complicated shapes and differ in their volume, surface area and membrane curvature. The ratio between the surface area of the cytosolic and luminal leaflets (*trans*-membrane area asymmetry (TAA)) determines the membrane curvature within different sites of the organelle. Thus, the shape of the organelle could be critically dependent on TAA. Here, using mathematical modeling and stereological measurements of TAA during fast transformation of organelle shapes, we present evidence that suggests that when organelle volume and surface area are constant, TAA can regulate transformation of the shape of the Golgi apparatus, endosomal multivesicular bodies, and microvilli of brush borders of kidney epithelial cells. Extraction of membrane curvature by small spheres, such as COPI-dependent vesicles within the Golgi (extraction of positive curvature), or by intraluminal vesicles within endosomes (extraction of negative curvature) controls the shape of these organelles. For instance, Golgi tubulation is critically dependent on the fusion of COPI vesicles with Golgi cisternae, and *vice versa*, for the extraction of membrane curvature into 50–60 nm vesicles, to induce transformation of Golgi tubules into cisternae. Also, formation of intraluminal ultra-small vesicles after fusion of endosomes allows equilibration of their TAA, volume and surface area. Finally, when microvilli of the brush border are broken into vesicles and microvilli fragments, TAA of these membranes remains the same as TAA of the microvilli. Thus, TAA has a significant role in transformation of organelle shape when other factors remain constant.

## 1. Introduction

Cells and membrane organelles are surrounded by the lipid bilayer membrane. The lipid bilayer can be easily bent and disrupted, but not compressed within the plane of the bilayer. The lipid bilayer can tolerate compression of the fatty acid chains, but cannot tolerate compression of the lipid heads along the direction lying within the plane of the bilayer [1].

Over recent years, it has been shown that lipids *per se* have significant roles in the determination of the shape of cellular organelles [1,2,3,4,5,6,7,8]. Indeed, spontaneous curvature and the expected shape of organelles can be computed directly from fluid lipid bilayers by modeling these observed bio-membrane shapes [8]. For example, after fusion of two spherical membrane compartments where one is highly curved, the newly formed spherical compartment needs to deal with the situation where the number of uncompressible lipid heads in the external leaflet is significantly higher than within the internal leaflet. As a result, intra-membrane stress appears within the region where there is an excess of lipid heads, and it lasts for some time [9] because the speed of transfer of lipids from one leaflet of the membrane to the other (with the help of flippases and floppases [10]) is relatively slow, whereas membrane transformations are extremely fast [11,12]. Thus, at least for some period of time, the intra-membrane stress will force the membrane to reshape, to release this stress.

**Figure 1:**
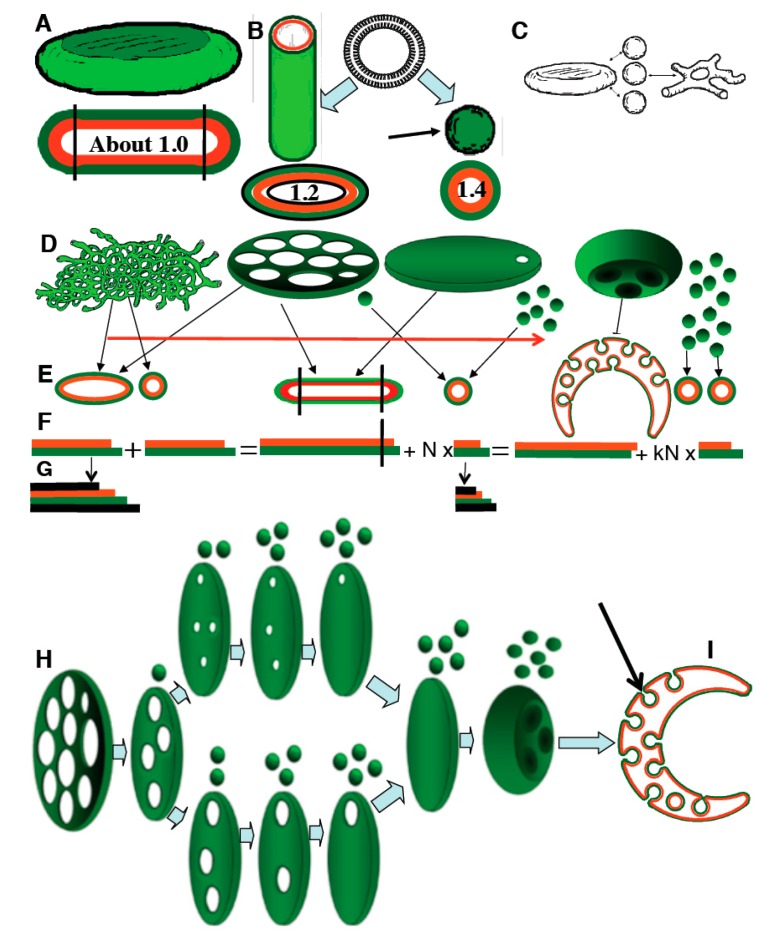
The model of the *trans-*membrane area asymmetry (TAA) redistribution. (**A**) Scheme of a lipid bilayer; (**B**) Planar lipid bilayer; (**C**) Closed, spherical lipid bilayer; (**A**–**C**) Different structural elements of the Golgi: disk (**A**), cylinder (**B**) and sphere (**B**, arrow). Assuming the bilayer thickness of 4 nm, the diameter of the sphere and the cylinder of 52 nm, and the thickness of the disk of 30 nm, the TAA values for the membrane disk (≈1.01), the cylinder (1.2), and the sphere (1.4) are indicated inside schemes showing a section of the corresponding geometrical figures; (**D**–**F**) Schematic representation of a Golgi tubular network transforming (from left to right) into a perforated disk and a few vesicles, and subsequently into an invaginated disk with internal buds and many vesicles; (**E**) Cross-sectional view of the structural elements of the Golgi. N and kN indicate the number of 52 nm vesicles. Orange, luminal monolayer; green, cytosolic monolayer; (**F**) TAA and the average ratio between the surface area of cytosolic (green) and luminal (orange) membrane leaflets remain constant in spite of the dramatic changes in GA morphology; (**G**) Scheme of the situation with membrane images after fixing of cells with OsO_4_, when atoms of osmium are precipitated on the external and internal surfaces of the lipid bilayer; (**H**) Two ways of transformation of a highly perforated disk during vesicle budding into a smooth disk, and ultimately, into an invaginated disk with internal buds (**H**, arrow); (**I**) The invaginated disk with internal buds has a TAA < 1.

The difference in the number of lipids in each membrane leaflet can be conveniently described in terms of the trans-membrane area asymmetry (TAA), which the ratio between the surface area of the cytosolic and luminal leaflet. TAA is closely related to the total curvature of the membrane [8]. In flat membrane parts, the curvature is zero, both leaflets have the same area and TAA = 1. In evaginated membranes the curvature is positive and TAA > 1, while in invaginated membranes the curvature is negative and TAA < 1 (Table S1). Hence, TAA is the largest in small vesicles (when the thickness of lipid bilayer is comparable in size with the diameter of spheres) where it can reach 1.4 (Figure 1A–F; Tables S1 and S2). In cylinders, one of the principal membrane curvatures is zero and thus typical cylinders have a twice as small total curvature as vesicles and TAA of approximately 1.2. The rims of large flattened compartments (e.g., Golgi cisternae) can be considered as bent half-cylinders, cut in the axial direction. On the other hand, the rims of small cisternal perforations are bent in two opposite directions and have a slightly lower total curvature and TAA than the outer rims, between 1 and 1.2. Finally, invaginated vesicles have a negative curvature and TAA as low as 0.7.

To test the idea that TAA can affect the shape of organelles is a particularly difficult task. Indeed, *in vitro* it is not possible to reach the concentration of proteins that exists within the cytosol, because proteins actively modulate the characteristics of lipid bilayers. However, to perform such experiments *in vivo* is even more difficult, because there is the need for an assay where it is possible to eliminate most of the other factors that can affect the shape of organelle membranes. 

One of the most suitable organelles to test the TAA hypothesis might be the Golgi apparatus (GA), the central station along the secretory pathway. The geometrical features of the GA are particular. The GA is composed of stacks of cisternae (*i.e.*, disks with toroidal rims that are often undulated). These disks have a thickness of 20 to 40 nm, and they contain perforations and are surrounded by a number of COPI-dependent vesicles, which are small 52 nm-diameter spheres [13,14,15,16,17,18,19,20]. The cisternae of the same or different stacks can be connected by tubules, which appear as straight or bent cylinders [21,22,23]. Thus, in the GA, the regions of relatively flat membranes of the flat cisternal sides coexist with the regions of highly curved membranes at the cisternal rims (which are often undulated), and with perforations and tubular extensions, thus providing a structure that is convenient for stereology measurements.

The shape of the GA is affected by frequent fusion–fission events that can change TAA, or be modulated *per se* by TAA. Several mechanisms might be responsible for the regulation of the GA TAA, and thus the shape of the GA: (1) external forces of intercisternal adhesion [6]; (2) activities of flippases or floppases (proteins that move lipid molecules from one leaflet to the other [10]), or other proteins or molecular machines that have similar effects; (3) lateral segregation of membrane lipids according to their preferred curvature, although this will have a small role because the decrease in the bending energy due to the segregation of the curvature-preferring membrane lipids into the cisternal rims is not large enough to drive extensive segregation [7]; and (4) extraction of highly curved membrane domains by COPI-dependent vesicles (our hypothesis). 

The development of three dimensional electron microscopy (EM), correlative light EM (CLEM) [24], and stereology [25] have enabled three-dimensional reconstructions of cellular organelles and have provided the tools for the measurement of membrane curvature. In the present study, we test the hypothesis that claims that if the volume and surface area of Golgi cisternae remain the same, the fast generation of small spheres from the cisternae will change their shape, because the flippases cannot compensate for the change in TAA. We present experimental evidence for this novel function of COPI vesicles; namely, for the extraction membrane curvature. Additionally, to demonstrate that this situation is not *ad hoc*, we demonstrated that according to the predictions of TAA, fusion of endosomal vesicles induces the formation of cytosol-oriented tubules and intraluminal vesicles, whereas fragmentation of the microvilli of epithelial cells of proximal kidney tubules occurs according to their TAA.

## 2. Results

To test whether TAA (in other words: curvature) has a significant role in the determination of shape of membrane organelles, we selected four different assays. The criteria for selection were the following: (1) Membrane transformation should be fast, or it should be possible to inhibit lipid flippases and floppases (*i.e.*, incubating cells or tissues on ice; see [26]); (2) Among the players participating in the fusion-fission events, there should be small spheres where TAA is high, and it should be possible to measure the overall curvature; (3) It should be possible to control the interchange of lipid in the membranes in the assay; namely, to isolate the membranes and prevent the delivery of new membranes or the departure of the existing ones.

When there is delivery or departure of membranes, it becomes almost impossible to demonstrate the role of TAA. It is necessary to develop an assay where the organelles have constant volume, surface area, and difference between the surface area of the cytosolic and extracellular leaflets of the surrounding membranes. Recent advances in EM have enabled three-dimensional reconstructions of cellular organelles, while the development of stereology has enabled the measurement of membrane curvature. One of the possibilities was to perform careful examination of organelle shape, volume and surface area with the help of stereological analysis adapted for EM examination [25].

For our experiments we selected: (1) Golgi disks with perforations along the rims, and COPI dependent vesicles; (2) endocytic spheres with a diameter of 200 nm or more, which are formed in a clathrin-dependent manner and can fuse with each other; and (3) cylindrical microvilli of the epithelial cells from proximal tubules in the kidney, which are preserved in K^+^-rich solution on ice [26]. 

On the basis of the literature for the speed of function of protein machines [10], we assumed (see Appendix) that the effects of the activity of putative (until now these proteins have not been identified) GA flippases (or similar proteins) and of ionic pumps that transfer ions between the lumen and the cytosol are slower than the effects of the physical mechanisms that drive the reorganization of lipid bilayers. Similar considerations should be correct for the second and third assays. In the case of the microvilli on ice, the flippases cannot work when lipid liquid crystals are hardened, or “frozen”, at low temperature. 

After development of the assays (see Experimental Section), with the help of the WOLFRAM MATHEMATICA software (available online: http://www.wolfram.com/; software dedicated to the estimation of complicated equations), we examined how the shapes of the organelles surrounded by lipid bilayers would behave after membrane fusion or fission, and when varicosities appear within the cylindrical membrane organelle. Our mathematical modeling told as that: (1) if volume, surface area, and the total TAA remain constant during the fusion of the disk surrounded by toroidal rims with a small sphere with higher curvature than that of the disk, disk undulations along the axis of the toroidal rim should appear; (2) In contrast, if the disk contains membranous pores that pass through the entire lumen, the effect would be different; namely, the widening of the pores; (3) For the opposite, if small spheres with high curvature are detached from the disks, the diameter of the pores should decrease; (4) Moreover, if the detachment of these spheres continues, the disk should lose its pores and the shape of the disk would be transformed into a figure with a shape like a deflated ball (Figure 1H,I).

In the case of fusion of two endocytic vesicles, *i.e.*, with a diameter of 120 nm, the resulting sphere should have a diameter of 143 nm and should also form a cylinder (tube) attached to the sphere, with a diameter of 43 nm and a length of 195 nm. If the diameter of the fusing sphere is about 200 nm and the tube is below 72 nm, the diameter of the resulting sphere should be 238 nm, and it should contain an internal bud (spherical invagination). If the diameter of the tube is >72 nm, an external bud (spherical evagination) should appear (see Appendix). In cells, the tubules growing from MVBs have diameters of <50 nm [27].

Thus, after the first fusion of two spherical 200 nm endosomes, the resulting endosome should contain only an external tubule. After the second round of fusion the invagination should appear, and so on. Finally, when the function of the flippases is significantly affected at low temperatures and the exchange of membranes is not possible, the formation of varicosites should induce immediate formation of constrictions, and in the case of their subsequent breakdown into vesicles, TAA of the membrane should not change.

On the basis of the literature on the relatively speeds of function of protein machines [10,11,12], we assumed (see Appendix) that the effects of the activity of putative (until now, these proteins have not been reported on the Golgi [10]) Golgi flippases and floppases (or similar proteins) and ionic pumps transferring ions between the lumen and the cytosol are slower than the effects of physical mechanisms driving the reorganization of lipid bilayers. As such, when the delivery of membranes to the isolated GA is blocked, total TAA, total volume and total surface area of the GA membranes (including stacks, tubules and COPI-dependent vesicles) should be constant (Figure 1H,I). Therefore, our mathematical analysis demonstrated that: (1) if the ARF/COPI machinery can form 52 nm vesicles while their fusion with the Golgi cisternae and tubules is blocked by inhibition of the SNARE machinery, extraction of TAA (curvature) from cisternae inside COPI vesicles would reduce TAA of the Golgi cisternae, initially leading to narrowing of the cisternal pores and then to invagination of the cisternal membranes; (2) if the ARF/COPI machinery are inhibited whereas the SNARE machinery remained active, the prevalence of the SNARE machinery would lead to consumption of 52 nm vesicles, and thus augmentation of TAA of Golgi cisternae and tubules. This would initially result in a widening of the cisternal pores and then to Golgi tubulation; (3) if both machineries were inhibited simultaneously and equally, the Golgi shape would not change; and (4) during all of these transformations, the total TAA and the ratio between surface area and volume of Golgi membranes would not change. This indicates that at the level of the GA, the disks and vesicles are in equilibrium with the tubular network (Figure 1C).

### 2.1. Role of the Trans-Membrane Area Asymmetry (TAA) in Golgi Reorganization

**Figure 2:**
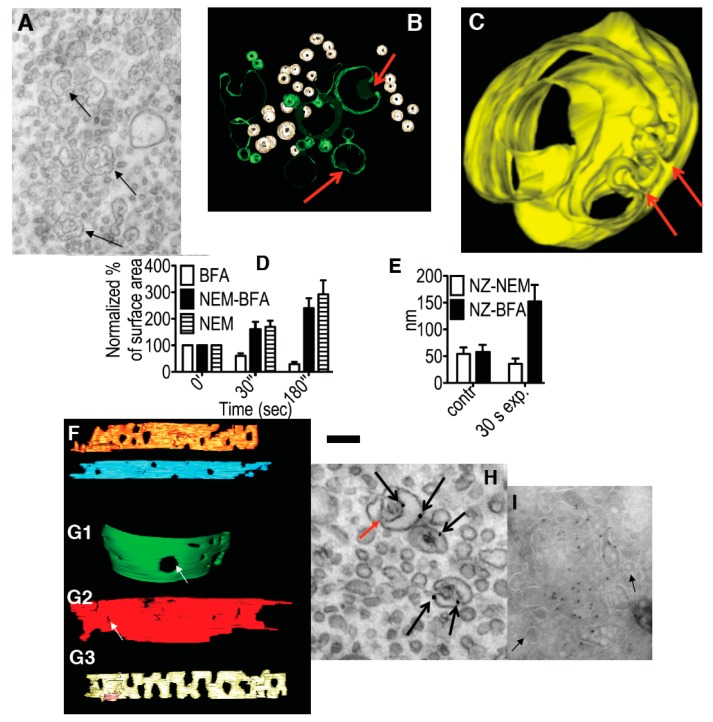
Effects of inhibition of membrane fusion on the Golgi. (**A**) HeLa cells 30 min after microinjection of αSNAPmu. Arrows, cisternal remnants; (**B**,**C**) Three-dimensional models of cisternal remnants after surface rendering. Arrows, invaginations. Cisternal remnants are green (**B**) and yellow (**C**). Vesicles in (**B**) are white; (**D**) Normalized surface areas of RPs *versus* Golgi cisternae after treatment with BFA alone (white bars), 30 s after treatment with *N*-ethylmaleimide (NEM) and then brefeldin A (BFA; black bars), or 30 s after NEM treatment alone (striated bars); (**E**) Mean diameter of cisternal pores determined using EM tomography. Each experiment was compared with its own control; (**F**) Cells were treated with 33 µM nocodazole (NZ) or 5 min, and then additionally incubated 30 s with NZ alone (blue cisterna in **F**), NZ + 5 µg/mL BFA (orange cisterna) or NZ + 1 µM NEM (yellow cisterna). (**F**,**G**) Examples of three-dimensional models of control (blue in **F**) penultimate medial cisternae after inhibition of vesiculation (NZ + BFA, orange in **F**) or membrane fusion (NZ + NEM, yellow in **F**) machineries; (**G**) Isolated Golgi membranes were incubated with 5 mg/mL native cytosol, the ATP restoration system (ARS), GTP: without (G1) or with the mutant of αSNAP (αSNAPmu; G2) for 20 min. Pores are indicated with white arrows. Addition of α-SNAPmu induced narrowing of cisternal pores; (**H**) HeLa cells treated with NEM and analyzed 3 min after re-warming; mannosidase II (ManII) was labeled by nano-gold (arrows). Red arrow, cisternal invagination; (**I**) Incubation of isolated Golgi membranes with native cytosol, ARS/GTP (**G**) and αSNAPmu for 90 min. Cryosection labeled for ManII. Vesicles (arrows) do not contain ManII. Bars. 300 nm (**A**); 100 nm (**B**); 50 nm (**C**); 250 nm (**F**,**G**); 120 nm (**H**,**I**).

To test the first prediction, we inhibited the SNARE machinery by microinjecting HeLa cells with a dominant-negative (L294A) His6-a-SNAP mutant (aSNAPmu). Analysis at the EM level 30 min after this microinjection showed enhanced vesiculation of the GA and the formation of invaginated cisternae (Figure 2A–C, arrows). To study earlier events after blocking membrane fusion, HeLa cells were treated on ice with NEM. Under these conditions, NEM inactivates the fusion factor NSF without affecting cell viability [28]. By 30 s after the wash-out of NEM and with re-warming of the cells, the number of COPI vesicles had increased (Figure 2D, striated bars). This vesiculation was accompanied by narrowing of the cisternal pores and smoothing of the cisternal rims (Figure 2F-yellow cisterna, Figure 2E-white bars) whereas after incubation with brefeldin A (BFA), the sizes of the cisternal pores increased (Figure 2F-orange, Figure 2E-black bars) in comparison with the control cells (Figure 2F-blue; Figure 2E, white bars).

By 3 min, the GA was almost completely vesiculated, and invaginated cisternal remnants appeared that labeled for ManII, which indicated that they originated from the Golgi cisternae (Figure 2H). As an independent approach, we used a well-established *in vitro* system based on isolated rat liver Golgi membranes. As shown in Figure 2G2 (compared with Figure 2G1 as control), after a 20 min incubation with native cytosol in the presence of an ATP regeneration system, GTP (ARS/GTP) and αSNAPmu, the mean cisternal pore diameter had clearly decreased, and by 90 min the cisternae were transformed into invaginated saccular structures that labeled for ManII and were surrounded by COPI vesicles (Figure 2I). Thus, inhibition of the SNARE machinery induces Golgi vesiculation, and it is accompanied by a narrowing of the cisternal pores that is followed by the generation of cisternal remnants with invaginations, in agreement with the TAA hypothesis. 

**Figure 3:**
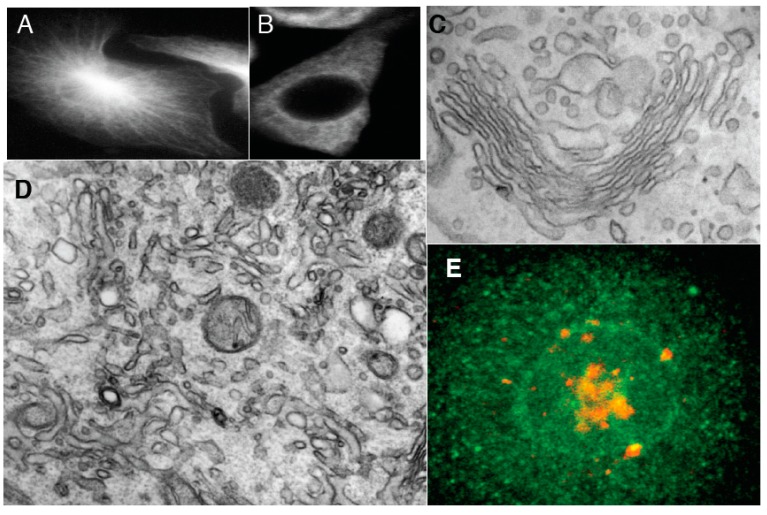

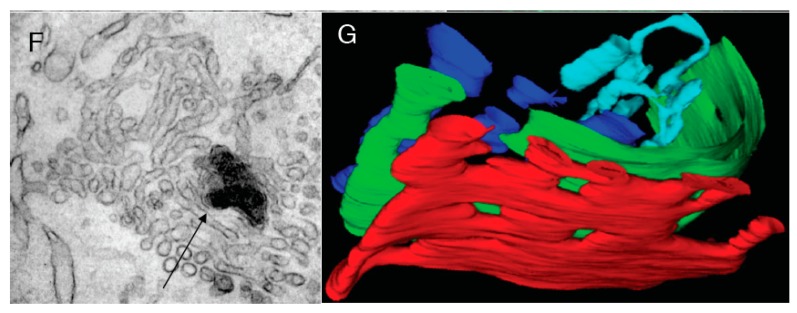
Control experiments. (**A**) Control HeLe cells labeled with an anti-tubulin antibody; (**B**) Cells treated with NZ; (**C**) Preservation of Golgi complex morphology in HeLa cells after 5 min NZ treatment; (**D**,**E**) BFA was added for 5 min after an irrelevant IgG (**D**) or the mutant of αSNAP (αSNAPmu; **E**) microinjection. Mannosidase II (ManII; red) did not redistribute into the endoplasmic reticulum, although most of the β-subunit of coatomer I (green) detached from the Golgi and diffusely labeled the cytosol; the Golgi stack was preserved; (**F**) Tubulated Golgi membranes after loading of cells with the WGA lectin conjugated with horse-radish peroxidase. The endosomal TGN is stained with OsO_4_ (arrow); (**G**) Three-dimensional tomogram reconstruction of an isolated Golgi stack. Bars: 10 µm (**A**,**B**); 150 nm (**C**,**D**); 5 µm (**E**); 100 nm (**F**,**G**).

**Figure 4:**
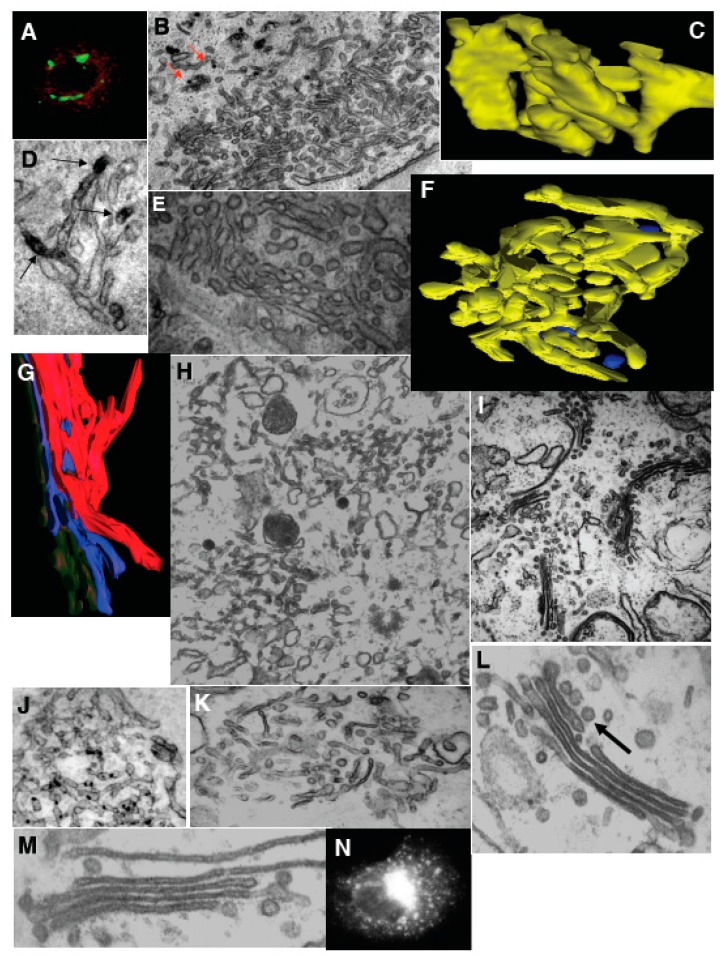
Transformation of Golgi stacks after inhibition of the ATP restoration system, coatomer I (COPI), and the SNARE-proteins. (**A**–**D**) HeLa cells treated with NZ (5 min) and then BFA + NZ (5 min); (**E**) GA of HeLa cells, 30 min after microinjection of an anti-βCOP antibody. (**A**) βCOP (red) and ManII (green) labeling; (**B**) labeling of β-COP (nano-gold-enhancement, red arrows); (**C**) Three-dimensional model of the GA; (**D**) immunoperoxidase labeling of ManII (arrows); (**E**) Routine EM section; (**F**–**N**) Inhibition of membrane fusion or ARF/COPI machine impairs GA shape in permeabilized cells. Ldl F cells were warmed to 40 °C for 6 h (**F**); SLO permeabilized (**G**,**H**); and incubated for 90 min at 32 °C with the ATP restoration system (ARS), GTP, plus native (**I**,**K**); COPI-depleted (**J**,**K**,**N**) or ARF-depleted (**M**) cytosol, with an anti-βCOP antibody (**K**), partially purified COPI cytosol (**K**,**N**), or mARF1 (**M**), and then prepared for EM. (**F**,**G**) Three-dimensional tomographic models; (**H**,**I**–**M**) Routine electron microscopic sections. (**N**) Immunofluorescence. Arrow in (**L**) shows the section of COPI vesicle. Bars, 10 µm (**A**,**N**); 400 nm (**B**,**H**,**I**–**K**); 70 nm (**C**,**G**); 100 (**F**); 120 nm (**D**,**E**,**L**,**M**).

To assess the validity of the second prediction, HeLa cells were treated with BFA, an inhibitor of ARFGEFs [29]. Simultaneously, to prevent the redistribution of the Golgi membranes into the endoplasmic reticulum under the action of BFA, microtubules were depolymerized using a short pretreatment (5 min) with nocodazole (NZ) before the incubation with BFA, for 0.5, 3, 5, or 20 min (in the continued presence of NZ to avoid microtubule re-polymerization). The short pre-treatment with NZ did not induce any visible changes in the GA morphology (Figure 3), but it clearly prevented BFA-induced redistribution of the Golgi enzyme ManII into the endoplasmic reticulum, although most of the βCOP had dissociated from the Golgi membranes within 5 min of adding BFA (Figure 4A).

At the EM level, at 0.5 min and, in particular, 3 min after BFA addition, the number of peri-Golgi vesicles had decreased, while the cisternal pores were enlarged (Figure 2G3,D: white bars). By 5 min, the cisternae were transformed into tubular networks, as seen by EM (Figure 4B) and electron tomography (Figure 4C). The tubulated membranes were labeled for the Golgi marker ManII (Figure 4D). The tubulated GA with WGA-HRP inside the lumen in the endosomal TGN is shown in Figure 3F. In an independent approach, the ARF1/COPI machinery was inhibited by microinjecting cells deprived of microtubules with anti-βCOP Abs; here the GA remained in the cell center, and again it was tubulated (Figure 4E). Thus, inhibition of the ARF/COPI machinery induces widening of the cisternal pores that is followed by Golgi tubulation.

To determine whether conversion of the tubulated GA into stacks is ARF/COPI specific using an independent approach that avoided drug treatments, we used mutant ldl F cells. These ldl F cells contain a temperature-sensitive version of ε-COP that is inactivated and completely degraded by an incubation at 40 °C for 6 h [30]. At the permissive temperature (34 °C), the Golgi stacks in these cells were normal, but after inactivation of εCOP as above, the GA was transformed into tubular networks (Figure 4F). If the ldl F cells were permeabilized after ε-COP inactivation and incubated with cytosol and ARS/GTP, the tubulated Golgi cisternae (Figure 4G,H) were converted back into flat and smooth cisternae that were surrounded by 52 nm vesicles (Figure 4I). This transformation occurred rapidly, and it was complete within 2 to 3 min. COPI-depleted cytosol (Figure 4J), ARF-depleted cytosol (Figure 3G), and incubation in the presence of an anti-βCOP antibody (Figure 4K) did not allow the Golgi stacks to reform. In contrast, after recomplementation of COPI-depleted cytosol with partially purified coatomer (Figure 4L), or of ARF-depleted cytosol with myristoylated ARF1 (Figure 4M), the Golgi stacks reformed normally and βCOP was concentrated again in the pericentriolar area (Figure 4N). Thus, the transformation of tubulated or highly perforated Golgi cisternae into more disk-like structures requires the extraction of membrane curvature into buds, varicose tubules, or vesicles by the ARF/COPI machinery, and this occurs at a striking speed and efficiency upon addition of coatomer I. 

**Figure 5:**
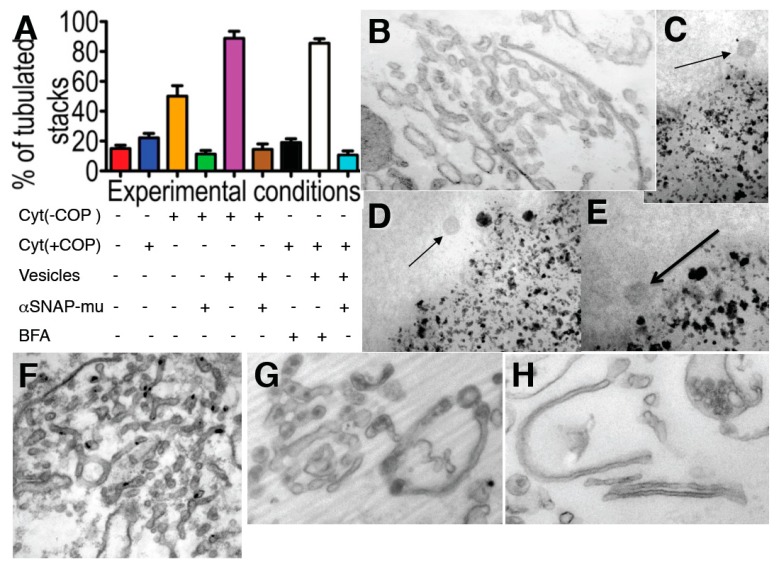

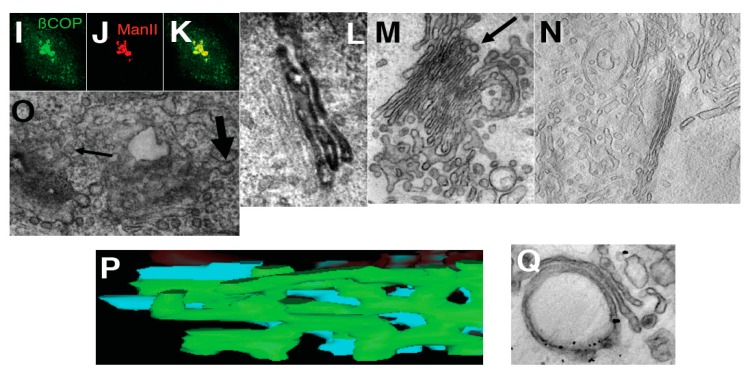
The Golgi tubulation induced by brefeldin A (BFA) depends on the availability of coatomer I-dependent (COPI) vesicles. (**A**) Incubation of isolated Golgi membranes for 90 min, always in the presence of the ARS, GTP, with the normal cytosol (Cyt (+COPI)), blue) or COPI-depleted cytosol: Cyt (−COPI)), without or with isolated COPI vesicles (Vesicles), the mutant of αSNAP (αSNAPmu) and/or BFA, as indicated below the graph. The degree of tubulation was estimated as indicated in Supplementary Materials. Red, low level of tubulation before incubation; (**B**) Incubation of Golgi membranes with COPI-depleted cytosol in the presence of ARS/GTP for 90 min transforms Golgi cisternae into tubules, although only partially (orange bar in **A**). Tubulation was not seen if αSNAPmu was added to the incubation medium (green bar in **A**); (**F**) Incubation of the Golgi membranes as in (**B**), but in the presence of isolated COPI vesicles (magenta bar in **A**). Higher degree of tubulation. Addition of αSNAPmu to incubation medium as in (**F**) blocked tubulation (brown bar in **A**); (**G**) BFA (2 µg/mL for 90 min) induced tubulation of isolated Golgi membranes in the presence of externally added COPI-dependent vesicles (white bar in **A**), and not in their absence (black bar in **A**), or when added together with the vesicles. When αSNAPmu was added, this tubulation was also blocked (**H**, light blue bar in **A**); (**C**–**E**) Isolation of COPI vesicles (arrows) isolated with the help of magnetic beads; (**I**–**K**) Revesibility of the changes in the Golgi shape. (**I**–**R**) HeLa cells were treated with NZ (5 min) and then with NZ + BFA (5 min), followed by NZ alone (5 min, **B**,**F**) and then BFA was washed out for 2 min (**I**–**K**,**N**) or 5 min (**L**,**M**,**O**), (**I**–**K**) Re-appearance of β-COP labeling (green) over ManII-positive structures (red) 2 min after BFA wash out; (**L**,**M**) Transformation of Golgi tubules back into polarized stacks of cisternae containing coated buds (thick arrow in **O**) and surrounded by 50–60 nm round profiles (arrows in **L**,**M**); (**N**) RPs visible 2 min after BFA washout were sections of varicose tubules; (**O**) Increase of the number of free 52 nm vesicles and COPI-coated buds; (**P**) Re-addition of BFA after reformation of GA (as in **L**) induced Golgi tubulation again; (**Q**) Re-appearance of stacked cisternae after treatment of isolated Golgi membranes with COPI-depleted cytosol and ARS/GTP for 90 min (see above), re-isolation of the tubulated Golgi membranes and additional treatment of these membrane with normal cytosol and ARS/GTP for a further 90 min. Bars, 200 nm (**B**); 100 nm (**C**,**D**); 80 nm (**E**); 200 nm (**F**,**M**,**N**); 150 nm (**G**,**H**,**L**,**Q**); 10 µm (**I**–**K**); 120 nm (**O**); 50 nm (**P**).

Next, we examined whether the membrane curvature that is stored in COPI-dependent vesicles is actually required for the tubular transformation of the GA. First, when isolated Golgi membranes were incubated with COPI-depleted cytosol in the presence of ARS/GTP for 90 min, the Golgi cisternae were transformed into tubular networks (in agreement with the results of Misteli and Warren [51]), although only partially (Figure 5A-orange bar; Figure 5B). Moreover, tubulation was not seen if αSNAPmu was added to the incubation medium (Figure 5A-green bar, Figure 5B). When COPI-dependent vesicles isolated using magnetic beads (Figure 5C–E, see [31]) (vesicles prepared according to Kweon *et al*. [28] and Yang *et al*. [32] gave identical results; our unpublished observations) were added to the incubation medium, the degree of Golgi tubulation greatly increased (Figure 5F, Figure 5A-magenta bar), and this stimulatory effect was blocked by αSNAPmu (Figure 5A-brown bar). Similarly, BFA (2 µg/mL for 90 min) induced tubulation of isolated Golgi membranes, although only in the presence of externally added COPI-dependent vesicles (Figure 5G, Figure 5A-white bar), and not in their absence (Figure 5A-black bar). This tubulation was also blocked by αSNAPmu (Figure 5H, Figure 5A-light blue bar). Thus, BFA-induced Golgi tubulation shows an absolute requirement for the fusion of COPI-dependent vesicles with the Golgi stack. We conclude that the fusion of COPI-dependent vesicles with the Golgi stack provides the curvature necessary for GA tubulation.

Importantly, during the initial period, the tubular transformation of the GA was fully reversible. If the cells were washed after 5-min BFA treatment (in the continued presence of NZ), the pericentriolar staining for βCOP re-appeared within 2 to 5 min (Figure 5I–K). Simultaneously, the Golgi tubules were transformed back into polarized stacks of cisternae (Figure 5L) that contained ManII (DAB precipitate) and coated buds, and were surrounded by 50–60 nm sized round profiles (RPs; Figure 5M). In the first 2 min after BFA washout, most of these RPs were derived from sections of varicose tubules (Figure 5N), but after 5 min, the number of free 52 nm vesicles increased three-fold (Figure 5O). If BFA was re-added to the medium 5 min after its washout, β-COP was once more released from the GA, and the GA became tubulated (Figure 5P). In a complementary approach, we incubated isolated Golgi membranes with COPI-depleted cytosol and ARS/GTP for 90 min (see above), re-isolated the tubulated Golgi membranes, and then incubated these with normal cytosol under the same conditions for a further 90 min. This incubation led to the re-appearance of stacked cisternae (Figure 5G). Thus, Golgi tubulation and stack formation are rapidly reversible via COPI-dependent vesicle formation and SNARE-dependent vesicle fusion.

**Figure 6:**
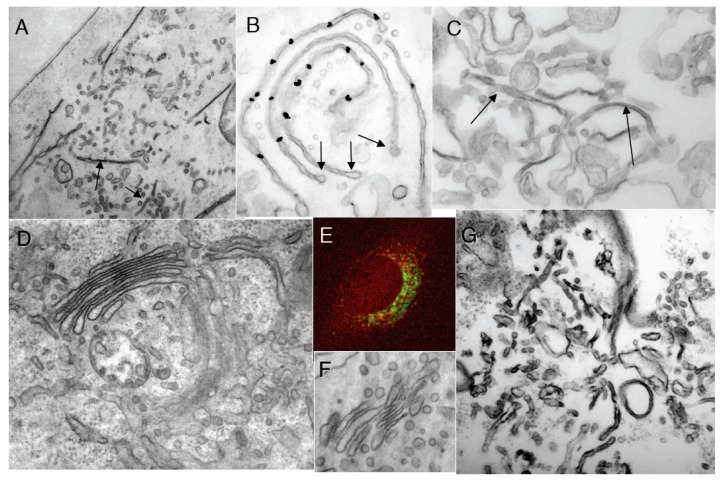

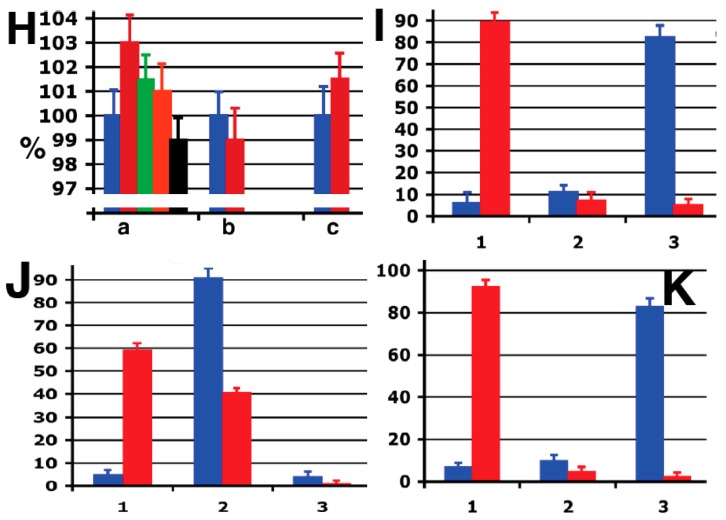
Protein machineries that are involved in GA shaping. (**A**,**B**) The ldl F cells were treated at 40 °C for 6 h, permeabilized with streptolysis O (SLO), then incubated with native cytosol, the ATP restoration system (ARS), GTP and a mixture of anti-GRASP55 and GRASP65 antibodies for 90 min, and prepared for electron microscopy (EM; **A**) or immuno-nano-gold-EM labeling for ManII (**B**). Arrows show non-stacked Golgi cisternae (**A**) or COPI-coated buds (**B**); (**C**) Isolated Golgi membranes were incubated with COPI-depleted cytosol and ARS/GTP and then re-isolated. Arrows show individual cisternae; (**D**,**E**) HeLa cells were microinjected with the mutants of αSNAP protein (αSNAPmu), then placed on ice for 10 min, treated with BFA for 30 min, and then prepared for EM (**D**) or immunofluorescence (**E**; anti-βCOP, red; anti-mannosidase II (ManII, green); (**F**) HeLa cells were microinjected with a mixture of αSNAPmu and an anti-β-COP antibody, and after 30 min prepared for EM; (**G**) Ldl F cells were treated at 40 °C for 6 h and permeabilized with SLO, and then incubated with COPI-depleted cytosol and ARS/GTP, plus αSNAPmu, for 90 min; (**H**–**K**) Quantification of mean Golgi membrane curvature; (**H**) Ratio between the cytosolic and luminal surface areas (a); integrated mean curvature (b); and tomographic estimation (c). Data were normalized with respect to the curvature 5 min after NZ treatment. Note: the *Y*-axis in **H** is shown with a break; (**I**–**K**) Distribution of surface area (blue bars) and TAA (red bars) between 50–60 nm RPs (1 in **H**–**K**), elongated profiles (2 in **B**–**D**) and cisternae (3 in **H**–**K**) after treatment of HeLa cells with NZ (5 min, **I**); NZ (5 min) and then NZ + BFA (5 min, **J**); or NZ (5 min), NZ + BFA (5 min) followed by BFA washout in the presence of NZ (5 min, **K**). For details on curvature quantification, see Supplementary Materials. Bars, 500 nm (**A**); 300 nm (**B**); 200 nm (**C**,**D**,**F**); 5 µm (**E**); 400 nm (**G**).

To examine the possibility that cisterna stacking contributes to the tubule-to-cisterna conversion, permeabilized ldl F cells (Figure 6A,B) with a pre-tubulated GA, or isolated Golgi membranes (tubulated by COPI-depleted cytosol and then re-isolated; Figure 6C), were incubated with cytosol and ARS/GTP in the presence of a mixture of antibodies directed against GRASP-55 and GRASP-65 that have been reported to almost completely inhibit Golgi stacking [34,35]. Under these conditions, the Golgi stacking was indeed strongly inhibited, but the efficiency with which tubules were converted into (isolated) cisternae, and the number of 50–60 nm buds/vesicles was not affected (Figure 6A–C). Collectively, these results show that the conversion of tubulated into smooth cisternal Golgi elements by the ARF/COPI machinery does not depend on the stacking of the Golgi cisternae.

To test the third prediction of the TAA redistribution model, we treated cells with BFA after microinjection of αSNAPmu to allow spreading of the mutant (Figure 6D), or with NEM. Under these conditions, the GA morphology was preserved (Figure 6D) and the Golgi enzymes did not redistribute into the endoplasmic reticulum, despite the normal inhibition of the ARF/COPI machinery by BFA (as seen by the diffuse cytosolic staining of βCOP; Figure 6E). When the cells were first treated with NEM, and BFA was added 20 s after re-warming, the increase in the number of RPs near the GA was halted, and they remained constant in number upon further incubation (3 min), while in control cells that were not treated with BFA, the number of RPs continued to increase (Figure 2D, striated bars). 

Similarly, microinjection of a mixture of an anti-β-COP antibody and αSNAPmu did not affect Golgi morphology for at least 40 min (Figure 6F), whereas when HeLa cells were microinjected with the anti-βCOP antibody alone, the Golgi cisternae were converted into a highly perforated and tubulated state (see Figure 4E). In addition, incubation of isolated Golgi membranes with a COPI-depleted cytosol in the presence of ARS/GTP and αSNAPmu did not result in GA tubulation or vesiculation (see Figure 5A-green bar). Finally, incubation of permeabilized ldl F cells (after heat-induced GA tubulation) with COPI-depleted cytosol in the presence of αSNAPmu did not affect the tubular state of the GA (Figure 6G). Thus, simultaneous inhibition of both the ARF/COPI and SNARE machineries stabilizes the GA morphology in any given state, in agreement with the TAA redistribution model.

The TAA redistribution model assumes that the total TAA and total volume of all of the structural elements of the GA are constant. To test this assumption, three different methods were used to estimate Golgi membrane curvature and volume. The following results were obtained for HeLa cells treated with NZ for 5 min: the ratio between the surface area of the cytosolic and the lum**i**nal leaflets, was 1.08 ± 0.01; EM tomography gave the ratio 1.09 ± 0.015; and the integral mean curvature was 331 ± 5 µm/µm^3^. The relative contribution of each structural element of the GA to the differences in surface area between the cytoplasmic and luminal membrane leaflets of the GA as a whole is given as the percentage TAA. The measured total TAA (see Table 1) of NZ-treated cells (mostly cisternae plus vesicles) was 1.08; the TAA for RPs of <60 nm diameter was 2.20 ± 0.02, for elongated profiles, 1.12 ± 0.03, and for cisternae, 1.01 ± 0.03. All of the other data were normalized with respect to the curvature 5 min after NZ treatment. All three of these methods showed that the total surface area of the Golgi membranes, the total GA volume, and the total (or mean) TAA did not change during GA tubulation or GA stack re-formation (Figure 6H–K). 

**Table 1:** Distribution of the *trans-*membrane area asymmetry (TAA) between the structural elements of the Golgi.

Experimental Design	Percentage Intersections			Percentage TAA		
	50–60 nm Round Profiles	Elongated Profiles	Cisternae	50–60 nm Round Profiles	Elongated Profiles	Cisternae
5 min; nocodazole (NZ)	7	10	83	92.5	5	2.5
5 min; brefeldin A (BFA)	5	91	4	59.2	40.7	0.1
5 min BFA, 5 min w/o BFA	6	11	82	89	7	5

Importantly, the distribution of TAA among the different structural elements of the GA (buds/vesicles, tubules, cisternae) changed dramatically during the shape transitions of the GA. Starting from a flat cisternal state with an accumulation of TAA in round profiles (buds/vesicles), BFA treatment resulted in a tubular state with more TAA in elongated profiles (tubules) and less in round profiles. BFA washout restored the flat cisternal state, with almost all of the TAA again concentrated in round profiles (Figure 6H–K).

In summary, we found that: (i) inhibition of the SNARE machinery alone reduced TAA of the Golgi cisternae and induced narrowing of the cisternal perforations, followed by invagination of cisternal membranes; (ii) inhibition of the ARF/COPI machinery alone increased TAA of the Golgi cisternae and induced widening of the cisternal perforations followed by GA tubulation; (iii) inhibition of both machineries did not change the GA shape significantly; and (iv) in all of these cases, TAA did not change significantly during the transformation of the GA shape, which suggested that the rapid interference with the ARF/COPI or SNARE machineries that results in dramatic changes in the morphology of the GA was not because of a change in overall TAA, but because of a redistribution of TAA among the structural elements of the GA.

### 2.2. Role of the Trans-Membrane Area Asymmetry in Reorganization of Microvilli of the Apical Plasma Membrane and Endosomes 

**Figure 7:**
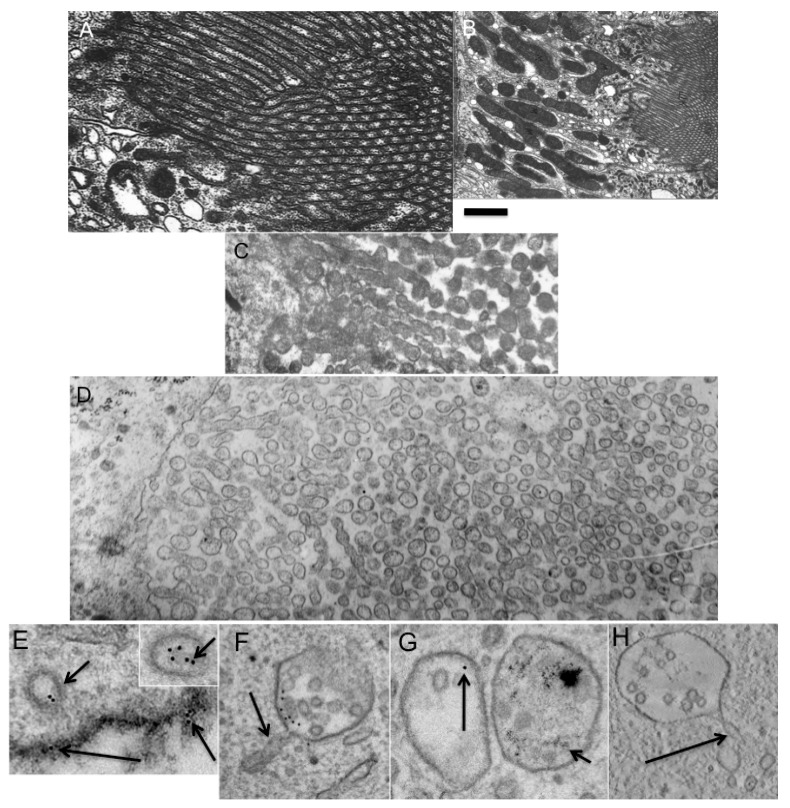
Breakdown of epithelial microvilli of kidney proximal tubule into membrane spheres. (**A**). Normal brush border consists of cylindrical protrusion of the apical plasma membrane; (**B**) General view of the brush border; (**C**) Formation of varicosite cylinders from cylindrical microvilli; (**D**) Transformation of varicose cylinders into small membrane spheres; (**E**) Formation of the endocytic clathrin-coated vesicles (short arrows) with a diameter of 90 and 128 nm (insertion on the right). The large vesicle contains more gold particles but no intraluminal vesicles. Long arrow shows gold particcles embedded into oxidized 3,3'–diaminobenzidine (dark precipitate); (**F**–**H**) Formation of multivesicular bodies containing tubular protrusions with a diameter of 40 nm (arrows in **F**,**H**). (**G**) Two multivesicular bodies: one filled with 5-nm gold particles (on the right, short arrow) and another filled with 10-nm gold particles (on the left, long arrow). Bars. 230 nm (**A**–**D**); 100 nm (**E**–**H**).

To demonstrate that the situation with the Golgi is not *ad hoc*, we performed additional experiments. A long time ago, the phenomenon of the breakdown of cylindrical microvilli of brush bodies of kidney epithelial cells into vesicles during the preservation of kidneys in an intracellular (*i.e.*, VNIIKIEX) solution was described [26]. The TAA hypothesis predicts that during this fragmentation of microvilli, TAA should remain the same. To test whether the TAA hypothesis is valid also for the conditions when the function of flippases is blocked by low temperature, we used our previous procedure for preservation of the kidney in the intercellular isotonic VNIIKIEX solution (see [26]). To this end, rat kidneys were removed from the bodies, perfused with the cold (0 °C) VNIIKIEX solution in 30 s, and examined under EM at 8 and 24 h after perfusion. In the control samples, the microvilli were normal just after the perfusion (Figure 7A,B). By 8 h, the microvilli were transformed into varicose tubules, while at 24 h, only vesicles were visible. This breakdown of the microvilli into spherical vesicles produced vesicles with a diameter of 198 ± 7 nm (Figure 7C,D). Measurement of TAA revealed that it was 1.13 immediately after the perfusion, and 1.18 after the breakdown of the microvilli into vesicles. If we take into consideration that during the treatment of the sample with OsO_4_ the thickness of the membrane increases from 4 to 8 nm, the real TAA was 1.083 and 1.085, respectively. Our calculations based on serial sections revealed that the surface area of the microvilli immediately after perfusion was equal to the surface area of the vesicles that formed from the microvilli. The ratio between the surface area and the volume decreased. The vesicles were almost perfectly spherical. This means that additional volume was delivered from the cellular bodies along the cytosolic continuity of the osmotic pressure into the vesicles, which increased in size when water went into them. Thus, the prediction of TAA claiming that under these conditions TAA would not change is confirmed.

To test whether the transformation of endosome shape during their fusion and maturation occurs in agreement of our mathematical model (see Appendix; Table S3), we prepared cells where endosomes were labeled with 5 nm and 10 nm gold (see Experimental Section). After such treatment, we observed three populations of endosomes inside the cells: newly formed; previously formed; and a small proportion of newly formed endosomes that fused with previously formed endosomes. The old endosomes contained only 5 nm gold, the newly formed endosomes contained only 10 nm gold (Figure 7E), while the endosomes that were newly formed by fusion with old endosomes contained both types of gold particles. After this, the cells were incubated at 37 °C for 5 min, and examined under EM (Figure 7F). As a control, we used samples that had been incubated with NEM, to block the fusion of endomembranes. We followed the fate of the endosomes, which were formed from the plasma membrane. These endosomes were labeled only with 10 nm gold. Their diameter was in the range of 100 to 130 nm. These endosomes could fuse with MVBs formed before the addition of 10 nm gold and labeled with 5 nm gold. In this case, we would see both 10 nm gold and 5 nm gold (Figure 7G). Thus, if an endomembrane structure contained 10 nm gold and 5 nm gold it was not examined. Only endosomes that contained only 10 nm gold particles were examined. These endosomes could not fuse with the plasma membrane, which was stabilized with oxidized DAB (Figure 7E) and a dilute concentration of 10 nm gold. In experimental cells, the number of gold particles and the mean diameter of the vacuoles that contained gold particles increased 3.2 ± 0.4-fold. Additionally many of these contained tubules that extended from the external membrane, and internal vacuoles and invaginations. Three-dimensional reconstruction revealed that the MVBs always contained not only intraluminal vesicles, but also external tubules with a diameter of 40 nm (Figure 7F,H). The mean diameter of vacuoles with gold was 240 ± 31 nm, whereas in cells that were not treated with NEM, the diameter was 160 ± 24 nm. The number of internal vesicles was about 2 per vacuole. Intraluminal vesicles were not found in MVBs with diameters <230 nm. In our samples, the ratio between the external (intraluminal) surface area and that internal (inside a vesicle) was 1.53. Endomembranes with a size >150 nm that contained only 10 nm gold had intraluminal vesicles and external tubules. TAA of MVBs with 10 nm gold revealed that it was equal to TAA of the 90–100 nm endosomes that contained 10 nm gold particles observed immediately before fusion. There was a positive correlation between the number of intraluminal vesicles and the size of the MVBs (*r* = 0.67). A similar correlation was seen between the number of tubular extensions and the number of intraluminal vesicles (*r* = 0.59). In contrast, after incubation in the presence of NEM, the mean number of gold particles inside vacuoles and the mean diameter of these vacuoles were not changed. Thus, fusion of spherical endosomes occurred according to the predictions derived from the TAA hypothesis.

## 3. Discussion

In the present study, we have tried to show that *in situ* under conditions where other factors participating in the shaping of cellular organelles are constant, such as volume and surface area of the membranes, TAA is involved in the determination of the shape of cellular membranes. The rationale for this study was the following. Initially, on the basis of known information about the shape of the GA, MVBs and microvilli of brush bodies, we modeled the transformations of selected organelles during the process of generation of highly curved small vesicles. These calculations were then used for the formulation of predictions within the area of organelle reshaping, which were not known. Then using our assays, we investigated these unexpected phenomena.

It could be said that while our hypothesis is interesting, it lacks a framework that can consistently address a number of Golgi situations and the assumptions that we have made. However, to support these mechanisms of membrane organelle reshaping that also involve intra-membrane tension due to the mismatch between the internal and external leaflets, we used not only the assay based on reshaping of the GA morphology during formation of consumption of COPI vesicles, but also the assays based on endosome fusion and the generation of highly curved intra-luminal vesicles of MVBs, and the transformation of the shape of microvilli (*i.e.*, their breakdown into small vesicles) of kidney epithelial cells of proximal tubules during their preservation in an intracellular solution on ice. Why did we select the behavior of the GA, the formation of MVBs, and microvilli breakdown for our analysis? Precisely because these structures can be relatively easily controlled in terms of the membrane delivery, and because in all of these cases there was the formation of small spheres that can extract significant amounts of curvature, *i.e.*, TAA. In the case of synaptic vesicles (*i.e.*, 42–44 nm vesicles), we could not design a suitable system here because it is very difficult to control the delivery of membrane to synapses. 

Furthermore, in all three of our assay systems, we used different approaches to minimize the involvement of flippases and other mechanisms whereby lipid molecules are transferred across the hydrophobic zone of the membranes. We selected examples of membrane reshaping that are either faster than the function of flippases or that occurred under conditions where flippases do not work, or work very slowly (*i.e.*, on ice). Additionally, we checked whether flippases are involved in the organelle reshaping by estimation of the mean TAA, and this was not the case. Moreover, it is well established that fusion of vesicles with membranes is significantly faster than the transport of lipids by flippases and floppases [10]. Again, to date, flippases and floppases that operate at the level of the GA have not been defined [10]. Thus, their effects would appear to be negligible here. 

We showed that formation of 52 nm COPI-dependent vesicles that extract positive curvature from Golgi membranes led to the reshaping of the Golgi membrane in agreement with the predictions derived from our modeling. We also found that fusion of spherical endosomes induced formation of MVBs only when the diameter of the endosomes was higher than the defined level, and that fusion induced the formation of thin tubules that protruded from the external membranes of MVBs. Finally, we demonstrated that during fragmentation of microvilli of brush borders of kidney epithelial cells, TAA of vesicles was equal to TAA of microvilli, as predicted by the TAA hypothesis.

At the level of the GA, these data provide evidence for a role of COPI vesicles as reservoirs for membrane curvature that can regulate the overall shape of the GA. Our data also explain why isolated Golgi membranes have a reduced sensitivity to BFA [36]. This occurs because isolated Golgi membranes do not include COPI vesicles [28]. We have also resolved a discrepancy between the results of Misteli and Warren [31], who described the tubulation of isolated Golgi membranes after inhibition of the ARF/COPI machinery, and of Happe *et al*. [37], who did not observe Golgi tubulation. In the experiments by Happe *et al*. [37], the Golgi cisternae were initially attached to mica, and in contrast to the Golgi preparation used by Misteli and Warren [31], COPI vesicles were almost certainly absent. Finally, during mitosis, the GA does vesiculate. Again, how is this explained other than *ad hoc*? During mitosis, not only are COPI-coated vesicles formed, but also mini-stacks remain [17], and the cisternae of these stacks are devoid of pores and often have onion-like shapes, as the TAA hypothesis predicts. 

Recently, it was shown [11,12] that after the fusion of a few synaptic vesicles with the pre-synaptic membrane, formation of hemispheres and buds that both had a diameter of 80 nm was observed, and that this phenomenon occurred much faster than it could do with clathrin coating, or with any other protein coating. The volume, surface area and TAA of such a hemisphere with a diameter (80 nm) twice that of synaptic vesicles (42–44 nm) are equal to the corresponding parameters of a synaptic vesicle. Similarly, the volume, surface area and TAA of a full membrane sphere with a diameter of 80 nm are equal to the corresponding parameters of two synaptic vesicles. Thus, the formation of one hemisphere reflects the fusion and integration of the membrane of one synaptic vesicle into the pre-synaptic membrane, whereas the formation of a bud with a clear neck reflects the fusion of two synaptic vesicles.

One of the explanations for this phenomenon could again lie in the TAA hypothesis. Fusion and consecutive integration of vesicular membrane into the planar pre-synaptic membrane leads to the augmentation of membrane tension, due to the increase in the difference between the surface areas of the cytosolic and external leaflets of the pre-synaptic membrane. This tension is based on the physical interactions between lipid molecules, and thus it is extremely fast. The pre-synaptic membrane tends to eliminate this tension by the induction of the rapid formation of invaginations. When the pre-synaptic membrane is stabilized by the actin network attached to the cytosolic surface of the membrane, invaginations are formed locally. This phenomenon could be explained by the following considerations: when the actin network exist, only areas not covered by actin are used for the formation of invaginations. In contrast, when the actin network is dissolved, the pre-synaptic membrane did not form local invaginations but changed its total curvature, which is not detectable at EM. However, this possibility was not assessed in the present study. Thus, in summary, we have provided here the first evidence for a role for TAA of lipid membranes in the regulation of the shape of the GA, endosomes, and protrusions of the apical plasma membrane *in situ*.

## 4. Experimental Section

Unless otherwise stated, all chemicals and reagents were obtained from the previously indicated sources [32,38,39,40,41,42] or from Sigma (Milan, Italy). Anti-βCOP (EAGE), anti-ManII, anti-membrin and anti-GOS28 polyclonal antibodies (pAbs) were purchased from Thermo Fisher Scientific Inc (Rockford, IL, USA), and all were used at a 1:500 dilution. Anti-giantin monoclonal Ab was from Abcam Inc (Cambridge, UK) and used at 1:1000 dilution. The anti-α-tubulin pAb was from Sigma (Milan, Italy), used at 1:500. The plasmid encoding aSNAPmu was a kind gift from R. Burgoyne (University of Liverpool, Liverpool, UK). Dynabeads with a diameter of 2.8 µm that are conjugated with M-280 sheep anti-mouse IgG were from Dynal Biotech ASA (Oslo, Norway). 

HeLa cells were grown as described previously [22]. The ldl F mutants of CHO cells (a kind gift from Dr. M. Krieger, Massachusetts Institute of Technology, Cambridge, USA) and wild-type CHO cells were cultured according to [30]. Preparation and microinjection of aSNAPmu and anti-βCOP was as described previously [28]. Microtubules were depolymerized as described previously [39]. Cells were treated with *N*-ethylmaleimide (NEM) as described, using 1 mM NEM. Ldl F cells were incubated at 40 °C for 6 h and permeabilized using a two-step streptolysin-O (SLO) permeabilization protocol described previously [28,32,43].

Golgi membranes and permeabilized ldl F cells were incubated for 90 min in a K^+^-rich transport buffer with 5 mg/mL normal cytosol, with ARF- or COPI-depleted cytosols, or with COPI-depleted cytosol that was then supplemented with an enriched COPI fraction, as prepared according to [28], and which contained 5 mg/mL native cytosol and ARS/GTP, without or with isolated COPI vesicles, and/or without or with 2 mg/mL αSNAPmu, as described previously [28]. The ratio between isolated Golgi membranes and isolated vesicles was 1:2. After the incubations, the samples were fixed with 1% glutaraldehyde, pelleted and prepared for EM. 

COPI-depleted cytosol, purified COPI, and recombinant ARF1 were prepared as described previously [44,45]. Golgi membranes were isolated from rat liver according to [4,28]. A Golgi vesicle fraction was isolated as described by [33] or [32]. Briefly, the vesicle fraction was isolated according to [28] and was incubated with anti-membrin and anti-GOS28 monoclonal antibodies for 1 h at 37 °C; then, 2.8-µm-diameter Dynabeads conjugated with M-280 sheep anti-mouse IgG (Dynal Biotech ASA, Oslo, Norway) were added directly to the vesicle fraction, and incubated at 4 °C for 1 h. The beads were then shifted to the lateral wall of the test-tube and collected using the magnetic device, or collected with the help of magnetic racks, according to the manufacturer instructions. Adsorbed vesicles were eluted with 50 mL UAPB (1.5 M urea: 0.02 M arginine: 0.008 M phosphate) at pH 9.0, and centrifuged (20,000× *g* for 90 min at 4 °C). Then the pellet was washed twice with K^+^-rich transport buffer. In all cases, to assess the purity of the vesicles isolated by different methods, the pellet obtained after intensive centrifugation was fixed with 0.05% glutaraldehyde plus 4% formaldehyde for 5 min and prepared for routine EM or immunoEM [28,32].

To study the breakdown of kidney microvilli, Wistar rats were used. The rats were obtained from the animal house of the Mario Negri Sud Institute, where the animals were inspected by the Veterinary Administrator of the Italian Ministry for Agriculture. The rats were handled according to the instructions described in [42]. They were anesthetized with ethyl ether, and the abdominal aorta was canulated. Then the vascular bed of the rat was perfused with a solution containing (per liter): 9 g K_2_SO_4_, 1 g KHCO_3_, 3.6 g MgSO_4_, 9 g sodium oxybutirate, 17 g glucose, 0.25 mL heparin, pH 7.4; VNIIKIEH solution; see [26]) for 5 min at 0 °C. Then the kidneys were removed from the body of the rat and places at 0 °C for 8, 24 or 48 h, and after this period they were immersed in 1% glutardehyde in 0.15 M HEPES (pH 7.2) for 1 h, and prepared for EM. 

To examine multivesicular bodies (MVBs), HeLa cells were incubated with cationized gold particles (diameter, 5 nm) for 10 min. Then the surface of the cells was carefully washed with the acidic (pH 6.0) VNIIKIEH solution (see above) at 0 °C for 5 min (under these conditions all of the gold particles were detached from the plasma membrane), and cells were then placed at 37 °C for 30 min to allow internalization of the cationized gold into MVBs. Next, the cells were placed in normal VNIIKIEH solution on ice, and EGF conjugated with 10 nm gold particles were added for 10 min. Then the cells were washed and placed in phosphate-buffered saline for 2 min at 37 °C, to allow internalization of the EGF-gold bound to their EGF receptors. By this time, round or oval profiles containing gold particles appeared in the cell cytoplasm. Next, the cells were placed in VNIIKIEH solution on ice and treated with wheat germ agglutinin (WGA) conjugated with horse-radish peroxidase (HRP), for 10 min [46]. Medium containing diaminobenzidine (DAB) and H_2_O_2_ was then added, and the cells were incubated for 20 min to oxidize the DAB and induce its polymerization in the plasma membrane and in all invaginations that preserved a direct luminal link with the extracellular space. Polymerized DAB attached to the plasma membrane blocked its ability to fuse with any organelle inside the cytoplasm. Then the cells were incubated in phosphate-buffered saline at 37 °C for 10 min. Finally, the cells were fixed in 1% glutaraldehyde, as described above, and prepared for EM. As a control, the cells were examined immediately after incubation at 37 °C. As a further control, some cells were placed on ice in the presence or absence of 1 µM NEM for 10 min, and after its washout with dithiothreitol [28].

Immunofluorescence microscopy, cell permeabilization [40], measurement of co-localization, conventional EM, three-dimensional reconstructions, correlative video-light EM, immunoperoxidase and nano-gold EM labeling, serial ultra-thin cryosectioning, counting of labeling density, and analyses by EM tomography were all carried out as described previously [22,32,41,47,48]. CLEM was performed as described previously [49]. Sections were examined in Tecnai-12 or Tecnai 20 transmission electron microscopes (FEI; Eindhoven, The Netherlands), and 30–40 micrographs at 36,000× magnification were taken for each sample. The micrographs were numbered and randomized for measurements of the areas encompassed by the Golgi membranes and the numbers of buds and vesicles with or without coating (coated or uncoated), as described previously [22,25,41].

The numbers of free 50–60 nm vesicles (when all of their three projections were round) and the sizes of the cisternal pores were measured using the EM tomography stack of images. We counted the numbers of real vesicles per side of a Golgi stack using vertical sections through the stack [25,32]. The estimation of the absolute volumes of the pericentriolar Golgi elements (V_G_) was performed as described previously [25], by counting the numbers of test points (P_G_) on the Golgi membranes using analysis software at a standard final magnification of 60,000×. To exclude the possibility that endosomal membranes were counted as Golgi membranes, the entire endosomal system was loaded for 30 min with WGA lectin conjugated with HRP, according to [46], and serial sectioning was applied, to determine whether a round profile belonged to the GA or the endosomal system. Additionally, intersections were classified into three classes: intersections with small (maximum width, <65 nm), intermediate (maximum width, >65 nm but <200 nm) and large (maximum width, >200 nm) structures. The following data were obtained for HeLa cells treated with nocodazole (NZ) for 5 min: GA volume—13.5 ± 0.33 µm^3^, surface area of luminal leaflet of Golgi membranes—1215 ± 26 µm^2^. The degree of tubulation of the isolated Golgi membranes was assessed on EM grids under 20,000× magnification, with the percentages of Golgi elements in tubulated and stacked states counted. A membrane aggregate labeled for mannosidase (Man)II with nano-gold was considered a Golgi stack if at least two straight profiles were attached to each other. If the membranes formed a clearly distinguishable tubular network, this was considered tubulated Golgi. All other cases were considered as unknown. 

To measure the sizes of cisternal pores, we examined five randomly chosen Golgi stacks sectioned vertically (when all membranes were visible) in sections passing through the centrosome or (in NZ-treated cells) through the center of a stack, judging from serial thick sections. The sizes of the cisternal pores were determined in tomograms at each time point. The surface density (with respect to the volume of the Golgi elements) (SA_V_G = k × I_cyt_/P_G_) was measured by a point-hit method, and the number of intersections of the cytosolic and luminal contours (I_cyt_ and I_lum_, respectively) of Golgi membranes using the left-low border of the standard test as the very thin test line. If a test line passed tangentially through the Golgi membranes, the intersections were evaluated at a higher magnification using magnifying glasses or the zoom function of the AnalySis software. The absolute area of the cytosolic and luminal membrane leaflets was estimated according to the formula SA_G_cyt = k × SA_V_G × V_G_ [50].

To measure membrane curvature, we applied three methods. The first was based on the estimation of the mean TAA, according to the formula TAA = I_cyt_/I_lum_. In another approach, the integrated mean curvature (M_v_) of the surfaces of the Golgi membranes was estimated according to Russ and Dehoff [50]. To this end, the test-grid of the dissector was placed over the images taken at a 43,000× magnification, and the number (N) of closed contours minus the number of holes inside them was counted inside the dissector grid, according to the dissector rule. M_v_ was estimated as M_v_ = 2 * pi × N_a_, where N_a_ = N/SA, and SA is the surface area of the dissector grid (Figure S1). To verify the first two methods, we used micrographs of COPI vesicles from the literature (*i.e.*, Figure 4A from [51]; Figures 7A and 8A from [52]; Figure 4B from [53]), and found that COPI vesicles have TAA of 2.58 ± 0.01, which is in good agreement with theory, taking into account that the thickness of biological membranes after osmification is about 9 nm [54]; the calculated TAA of 52 nm vesicles with 9 nm thick bilayer would be 2.5 (Figure 1G). We measure the thickness of the membranes along the secretory pathway after our preparation of samples, and found that it was 8.6 nm. Note that the curvature measurements were performed with high precision (standard error, <1%). Then, TAA was counted again taking into consideration that the membranes after treatment with OsO_4_ are twice the thicker.

Estimations of TAA, surface area and volume were performed on the basis of EM tomograms according to Vanhecke *et al*. [55]. The surface areas of two surfaces of Golgi membranes were rendered using the IMOD software, according to Ladinsky *et al*. [13]. Volume, surface area and TAA we also measured on serial sections of centrally located microvilli of the brush body of proximal epithelial cells, with estimation of the percentages of MVBs containing intraluminal vesicles and 10 nm gold, but not 5 nm gold, inside the internal lumen. After generation of virtual slides from EM tomography, the three projections of the same round profile visible on the face projection were viewed. We considered a round profile (inside red circle) as an actual vesicle only when all three of these projections were round profiles. The following softwares were used: IMOD, for alignment of images, and ImageJ, to adjust contrast and brightness in images. The data presented are from at least three independent experiments, each performed in duplicate. At each experimental point, 25 cells were randomly selected and serially sectioned through the pericentriolar area. The percentage of cells containing Golgi stacks in the pericentriolar area was also estimated. The randomized data were entered into a computer plotting program (PRISM) and sorted, with the various parameters computed for each sample.

## 5. Conclusions

Thus, when organelle volume and surface area are constant, TAA can regulate transformation of the shape of different membrane organelles: for instant, the Golgi apparatus, endosomal multivesicular bodies, and microvilli of brush borders of kidney epithelial cells. Extraction of membrane curvature by small spheres, such as COPI-dependent vesicles within the Golgi (extraction of positive curvature), or by intraluminal vesicles within endosomes (extraction of negative curvature) changes the shape of these organelles. 

## Supplementary Materials

Supplementary materials can be found at: http://www.mdpi.com/1422-0067/16/03/5299/s1.

## Abbreviations

COP: coatomer protein
EM: electron microscopy
GA: Golgi apparatus
Man: mannosidase
NZ: nocodazole
SNAP: soluble NSF attachment protein
TAA: *trans*-membrane area asymmetry

## Appendix

### Theoretical Background

Here, we present a theoretical model that describes the transformation of a Golgi cisterna from a disk-like state into a tubular state, without changes in total membrane surface area, total TAA, or total internal volume.

In reality, a Golgi cisterna represents a disk with a toroidal and slightly undulated rim (Ladinsky *et al*. [13]). On the other hand, from our tomography data, it is clear that Golgi tubules induced by BFA treatment do not represent simple cylinders, but rather branched tubules with some flattened zones at the branching points. Finally, we demonstrated that upon BFA washout, this tubular network quickly (within 2 min) transforms into cisternae and buds/vesicles, restoring its original shape as detected before the administration of BFA. Using these phenomena we will perform some mathematical modeling.

General parameters used in formulae:

∆—difference;
SA—surface area;
V—volume;
pi—the ratio of the circle circumference to its diameter;
*—multiplication;
/—division;
^2—the square of a number
^3—the cube of a number

### The Simplest Method of Modeling

If the cisternal and pore diameters are much larger than the cisternal thickness, the overall trans-membrane asymmetry (∆SA or TAA) is proportional to the surface area (SA) of the strongly curved membrane, which is in turn proportional to the cisternal perimeter + the total perimeter of the pores (openings). We assume that in the Golgi cisternae rim, the curvature is much rgeater than that in the central part of the cisternae [8]. Therefore, as a first approximation, there is no difference between the rim undulations and the pores.

∆SA = h × SArim/Rt
(1)

where h is the distance between the external and internal surfaces of a leaflet in the bilayer and Rt is the rim curvature.

SArim is the surface area of the rim, and is given by:

SArim = L × pi × Rt
(2)

where L is the rim length, p x Rt is the arc length of the rim cross section.

Using these simple equations, both cisternal pores and all kinds of the cisternal rims are covered (for example in the case of bulbous rims, Equation (2) is simply SArim = L × 4/3 ×pi × Rt). In the case of circular cisternal pores, L is 2 × pi × Rp, and in the case of an undulated rim, L is simply the fractal length of the rim. In experiments, only Rt and the total cisternal L would have to be measured, without linking the measurements directly to a particular predefined cisternal geometry.

Budding and fission of a small sphere (vesicle) from a flattened tube or disk with a toroidal rim is accompanied by extraction of more surface area from the external leaflet than from the internal leaflet. The surface area of the external leaflet of a typical COPI vesicle is 0.008495 µm^2^, while the internal (luminal) leaflet measures 0.006082 µm^2^. Thus, the formation of one COPI vesicle typically extracts 0.002413 µm^2^ more surface area from the external than from the internal membrane leaflet, decreasing the difference between eSAT and iSA. The system will respond to the relative loss of external surface area by diminishing the level of rim undulation (F), and by decreasing the radius (Rp) of the pores, or by the formation of invaginated structures, like an invaginated semi-sphere.

Smoothing of the cylindrical rim from the 2nd level (F = pi^2/4) to the 1st level (F = pi/2) or from the first level (F = pi/2) to the zero level (F = 1) of undulation would be sufficient to compensate for the detachment of 75 spheres with diameter of 52 nm for 1 µm of the rim length of a cylindrical rim with a 26 nm radius. On the other hand, when the disk of a flattened tube reaches the first level of undulation, the second step of compensation after the detachment of 52 nm spheres would be to decrease the diameters of the openings. Assuming there is no change in the level of rim undulation, the narrowing of two pores from Ro = 0.1 µm to Ro = 0.015 µm would compensate for the detachment of approximately five 52 nm vesicles.

Finally, if the difference in surface area between the two membrane leaflets, namely, the external and internal (eSA/iSA or TAA), is consumed by vesicle detachment when the openings have almost closed and the rims are smooth, a further detachment of vesicles would result in an invaginated structure in which TAA is smaller than TAA of the simple disk with a toroidal rim or a flattened tube. In all of these cases, we assumed that there is no delivery of new membranes and there is no exchange of volume with another structure (Figure 1).

Thus, this method suggests that the extraction of curvature by COPI vesicles would lead to the disappearance of undulated rims or diminution of pore diameters. In contrast, addition of curvature due to fusion of the cisternae with COPI vesicles would lead to augmentation of pore size or undulation of rims.

### The Second Method

For this type of calculations, it is practical to examine transformations of only one Golgi cisterna, which can be represented as a flat disk with a toroidal rim. In general, our modeling demonstrated that addition of curvature to simple disks with toroidal rims should be compensated for by formation of pores and an increase in their size, or by augmentation of the level of rim undulation. However, in reality, we do not observe a significant level of undulation along cisternal rims. Moreover, undulation of rims does not affect the resulting TAA; the single problem here is the increase in the surface area localized within the undulated rims. Therefore, here, we did not examine the role of rim undulation in the shaping of the GA, and we focused our attention mostly on the role of pores in the modulation of the shape of the Golgi cisternae.

### Description of Shapes

#### 1. Sphere:

Parameters for a sphere:

Rs—external radius;
d—bilayer thickness;
N—number of spheres

Internal SA of a sphere (iSAs) = 4 × pi * (Rs − d)^2
(3)

External surface area of a sphere (eSAs) = 4 × pi × Rs^2
(4)

Internal volume of a sphere (iVs) = 4/3 × pi × (Rs − d)^3
(5)

#### 2. Invaginated semi-sphere:

Peculiar parameters for solid invaginated semi-sphere with toroidal rim:

Riss—main radius;
2 × Hiss—thickness of semi-sphere;
Restrictions: 0 < d < Hiss << Riss

Internal surface area of solid invaginated semi-sphere (iSAsiss) = 2 × pi^2 × Riss × (Hiss − d) + 2 × pi × ((Riss + Hiss − d)^2+(Riss − Hiss + d)^2)
(6)

External surface area of solid invaginated semi-sphere (eSAsiss) = 2 × pi^2 × Riss × Hiss + 2 * pi × ((Riss + Hiss)^2 + (Riss − Hiss)^2);
(7)

Internal volume of solid invaginated semi-sphere (iVsiss) = pi^2 × Riss × (Hiss − d)^2 + 2/3 × pi × ((Riss + Hiss − d)^3 − (Riss − Hiss + d)^3)
(8)

#### 3. Solid disk:

Parameters for a disk with a toroidal rim:

Rsd—radius of the flat part;
2 * Hsd—thickness;
d—bilayer thickness;
Restrictions: 0 < d < Hsd < Rsd

Internal surface area of solid disk (iSAsd) = 2 × pi × Rsd^2 + 2 × pi^2 × (Hsd − d) × Rsd + 4 × pi × (Hsd − d)^2
(9)

External surface area of solid disk (eSAsd) = 2 × pi^2 × Rsd × Hsd + 2 × pi × ((Rsd + Hsd)^2 + 4 × pi × (Rsd − Hsd)^2)
(10)

Internal volume of solid disk (iVsd) = 2 × pi × Rsd^2 × (Hsd − d) + pi^2 × (Hsd − d)^2 × Rsd + 4/3 × pi × (Hsd − d)^3
(11)

#### 4. Perforated disk with a toroidal rim with unequal openings also with toroidal rims:

Parameters for disk with toroidal rim with k1 openings of radius B1 and k2 openings of radius B2:

Rpd—radius of the flat part of perforated disk;
2 * Hpd—thickness of perforated disk;
k—number of openings;
B—flat radius of an opening;
Restrictions: 0 < d < Hpd < B < Rpd

Internal surface area of perforate disk (iSApd) = 2 × pi × (Rpd^2 − k1 * B1^2 − k2 × B2^2) + 2 × pi^2 × (Hpd − d) × (Rpd + k1 × B1 + k2 × B2) + 4 * pi * (Hpd − d)^2 × (1 − k1 − k2)
(12)

External surface area of perforated disk (eSApd) = 2 × pi × (Rpd^2 − k1 × B1^2 − k2 × B2^2) + 2 * pi^2 * Hpd * (Rpd + k1 * B1 + k2 * B2) + 4 × pi × Hpd^2 × (1 −k1 − k2)
(13)

Internal volume of perforated disk (iVpd) = 2 × pi × (Hpd − d) × (Rpd^2 − k1 × B1^2 − k2 × B2^2) + pi^2 × (Hpd − d)^2 × (Rpd + k1 × B1 + k2 × B2) + 4/3 × pi × (Hpd − d)^3 × (1 − k1 − k2)
(14)

Assuming that k2 = 0, the last set of formulae can be easily transformed into formulae describing a perforated disk with a toroidal rim with equal openings also with toroidal rims. Therefore these formulae are not shown.

### Stereometrical Transformations

Here, we use simplified shapes that mimic the GA to model the second stage of these transformations, namely, the transformation of a perforated Golgi disk with several round openings into a solid disk (with no openings) and a collection of small spheres, to see if the resulting estimates are consistent with the TAA hypothesis.

We perform this analysis by computing the external and internal surface areas and the volumes enclosed by the figures involved in the transformation, while trying to find figures for the remaining unknowns, such that that the difference between the sum of iSA1, or eSA1 or iV1 of the figure(s) 1 before transformation and the sum of iSA2, or eSA2, or iV2 of the figure(s) 2 after our transformation should be zero. For instance, when the perforated disk PD with k equal openings is transformed into a solid disk and N spheres, the following equations should be valid:

∆iSA = iSApd − iSAsd − N × iSAs = 0
(15)

∆eSA = eSApd − eSAsd − N × eSAs = 0
(16)

∆iV = iVpd − iVsd + N × iVs = 0
(17)

Here; we have three nonlinear Equations (13)–(15) that involve nine unknowns; namely; Rs; N; Rsd; Hsd; k; B; Rpd; Hpd; that are subjected to additional constraints 0 < d < Rs; 0 < d < Hsd < Rsd and 0 < d < Hpd < B < Rpd; and moreover; k and N must be non-negative integers.

If a perforated disk containing k1 openings with radius B1 undergoes fusion with N spheres and is transformed into a perforated disk containing k2 openings with radius B2, we have three similar equations:

∆iSA = iSApd2 − iSApd1 − N × iSAs = 0
(18)

∆eSA = eSApd2 − eSApd1 − N × eSAs = 0
(19)

∆iV = iVpd2 − iVpd1 + N × iVs = 0
(20)

The number of unknowns increases, namely: Rs, N, Rpd1, Hpd1, k1, B1, Rpd2, Hpd2, k2, B2. The constraints remain almost the same: 0 < d < Rs; 0 < d < Hpd1 < B1 < Rpd1; 0 < d < Hpd2 < B2 < Rpd2. Additionally, k1, k2 and N must be non-negative integers.

Therefore, we gave defined numbers to some unknowns. These numbers were derived from parameters experimentally observed for Golgi cisternae and COPI vesicles. For instance, we always consider in our modeling that d is fixed and equal to 4 nm.

### First Transformation

A perforated disk with k1 opening (Hpd1, Rpd1, d, B1, k1) plus N small spheres (Rs, d) are transformed into a perforated disk with k2 openings (Hpd2, Rpd2, d, B2, K2). The following quantities, namely, iSA, eSA and iV, should not change during the transformation (see above).

To minimize the number of unknowns we assigned the following values to the unknowns: d = 4.0; k1 = 1.0; Rs = 25; Hpd1 = 34.0; Rpd1 = 450.00; *n* = 8; k3 = 3. These calculations were performed using the standard nonlinear minimization package in the Mathematica software.

The calculation gave the following numbers: Hpd2 = 35.3066; Rpd2 = 472.866; B2 = 137.168 and the errors were small (see below).

This indicates that when iSA, eSA and iV remain constant, fusion of several small spheres with a larger solid disk forces the solid disk to acquire perforation(s). In an opposite way, when several small spheres are detached from a perforated disk, the openings in the disk should disappear and the disk becomes solid.

### Second Tranformation

A solid disk with toroidal rim with k openings (Hsd, Rsd, d, B, k) is transformed into a solid invaginated semisphere with toroidal rims (Hiss, Riss, d) plus N small spheres (Rs, d). iSA, eSA and iV should not change during the transformartion (see above). To minimize the number of unknowns, we assigned the following values to the unknowns: d = 4.0; N = 5.0; k = 1.0; Rs = 25; Hiss = 34.0; Rpd = 450.00. Calculation with the help of the Mathematica software gave the following numbers: Hpd = 28.3901; Riss = 326.29; B = 109.868 (Table S2).

Thus, detachment of small spheres from a solid disk with a toroidal rim transforms the disk into an invaginated semi-sphere.

### Third Transformation

A perforated disk with a height of 70 nm (Hpd = 35 nm) and a radius Rpd = 500 nm, which contains three openings (k = 3), with the radius of each opening B1 = 190 nm, undergoes transformation into a solid disk plus N spheres with a diameter of 50 nm. We minimized the sum of the absolute relative differences of the total external and internal areas and the total enclosed volumes before and after the transformation subject to the constraints above and obtained the following solutions. The resulting solid disk must have a radius Rsd = 430 nm, and a thickness 65.2 nm (Hsd = 32.6 nm); the number of spheres produced would be *n* = 20.

This indicates that fusion of small spheres with a larger perforated disk induces the formation of openings inside the disk. In the opposite way, this indicates that when several small spheres are detached from a perforated disk, the openings inside the disk should disappear.

### Relative Errors Obtained for the Transfromations

We checked how good the approximations are by computing three relative errors: internal and external areas, and volume. Since N was constrained to nonnegative integers, we evaluated the absolute relative differences of the total external and internal area and the total enclosed volume for the minimizing solution. The errors are relatively small. For instance, the first transformation gave the following errors.

RE(iSA) = 0.3%
RE(eSA) = 0.6%
RE(iVi)= 0.1%,

In all other cases, the errors were lower.

Thus, we obtained relatively good fits to the three equations that describe iSA, eSA and iV. We were also pleased to see that the parameters of the minimizing solution were in the experimentally observed ranges.

### Role of Decrease in Internal Volume on TAA

Here, we formulated the final prediction derived from the TAA model. We compared alterations in TAA after a decrease of the volume of the disk with a toroidal rim. Let us assume that the diameter of the disk is 1 µm and the height is 40 nm. In this case, the surface area of the external leaflet will be 1,772,024 nm^2^, whereas of the internal leaflet is 1,730,363 nm^2^. TAA is thus 1.0240763. If we decrease the volume of the disk and assume that its height is 20 nm and that the surface area of the external leaflet is constant, the diameter of the thin disk will be 515.6 nm, the surface area of the external leaflet of this thin disk will be 1,772,325 nm^2^ and the TAA will be 1.0239374. There is no statistically significant change in TAA. Thus, a decrease in the volume of the Golgi disks will not induce changes in their TAA.

In summary, our models predict that budding and fission of COPI vesicles transforms a highly perforated Golgi cisterna with undulated rim into a cisterna with narrower openings and less undulated rims. When the level of undulation became minimal, further detachment of COPI vesicles transforms a solid disk into a solid invaginated semi-sphere. Similarly, the fusion of COPI-dependent vesicles with a Golgi cisterna would be expected to widen cisternal pores and increase the level of rim undulation. Finally, a decrease in internal volume does not change TAA, and does not induce significant alterations in the shape of the GA.

### Endosomes

**Case 1: All three geometric parameters (*V, A*, ∆*A*) are conserved.**

When two spheres fuse and form a new sphere with a different radius, and a cylinder with radius Rc and length H, there is actually a solution even if all three geometric parameters are conserved (it is not an easy solution—it was found by the Mathematica programme). Note that TAA is an intensive parameter and it is not necessarily conserved; e.g., TAA of one sphere is 1.04, and TAA of two equal spheres remains 1.04. The approximate solutions are (here Rs is the radius of the initial two spheres):

Radius of the resulting sphere: Rs2 = 1.18959 Rs
Radius of the resulting cylinder: Rc = 0.360851 Rs
Length of the resulting cylinder: H = 3.24164 Rs

For example

In the case, where the radius of the two fusing spheres is 100 nm, the solution is:
Rs2 = 119 nm, Rc = 36 nm, H = 324 nm.
In the case, where the radius of the two fusing spheres is 120 nm, the solution is:
Rs2 = 143 nm, Rc = 43 nm, H = 389 nm.

**Case 2: All three geometric parameters (*V, A*, ∆*A*) are conserved and in addition the radius of the resulting cylinder is fixed.**

If in addition to Case 1, the radius of the cylinder is fixed (e.g., Rc = 20 nm), the geometric parameters can be conserved only if a third sphere emerges (Figure S2). If the fixed radius of the cylinder is larger than that determined in Case 1, the resulting extra sphere would be an evagination. And *vice versa*, if the fixed radius of the cylinder is smaller than that determined in Case 1, the resulting extra sphere will be an invagination. For example:

Rc = 40 nm: Rs2 = 118 nm, H = 304 nm, Rs3 = 6 nm (the third sphere is an evagination)
Rc = 20 nm: Rs2 = 124 nm, H = 406 nm, Rs3 = 25 nm (the third sphere is an invagination)

### Examples:

1. When 10 vesicles with diameter $D_{0}=200\text{ nm}$ fuse, the result is a sphere with diameter $D_{s}\approx 1.8\times D_{0}=360\text{ nm}$, and a cylinder with diameter $D_{c}\approx 0.43\times D_{0}=86\text{ nm}$ and length $L_{c}\approx 16\times D_{0}=3200\text{ nm}$.

2. The diameter of the resulting sphere is twice the diameter of the fusing vesicles ($D_{s}=2D_{0}$) when approximately n=15 vesicles fuse. The length of the cylinder at that point is approximately $L_{c}\approx 26\times D_{0}$ while the diameter of the cylinder remains at approximately 43% of $D_{0}$.

3. However, there could be the second variant, when each consecutive fusion between vesicles would give a new external tubule according to the regularities described in the first example. And if the diameter of this tubule is lower than 40 nm, there should also be the generation of a new intra-luminal vesicle. Thus, there could be a correlation between the diameter of the resulting sphere and the number of thin tubules, and between the diameter of the sphere and the number of intra-luminal vesicles.

## References

[1] Kozlov M.M., Campelo F., Liska N., Chernomordik L.V., Marrink S.J., McMahon H.T. (2014). Mechanisms shaping cell membranes. Curr. Opin. Cell Biol..

[2] Lee H.J., Peterson E.L., Phillips R., Klug W.S., Wiggins P.A. (2008). Membrane shape as a reporter for applied forces. Proc. Natl. Acad. Sci. USA.

[3] Weigert R., Colanzi A., Mironov A., Buccione R., Cericola C., Sciulli M.G., Santini G., Flati S., Fusella A., Donaldson J.G. (1997). Characterization of chemical inhibitors of brefeldin A-activated mono-ADP-ribosylation. J. Biol. Chem..

[4] Weigert R., Silletta M.G., Spanò S., Turacchio G., Cericola C., Colanzi A., Mancini R., Polishchuk E.V., Salmona M., Facchiano F. (1999). CtBP/BARS induces fission of Golgi membranes by acylating lysophosphatidic acid. Nature.

[5] Beznoussenko G.V., Dolgikh V.V., Seliverstova E.V., Semenov P.B., Tokarev Y.S., Trucco A., Micaroni M., di Giandomenico D., Auinger P., Senderskiy I.V. (2007). Analogs of the Golgi complex in microsporidia: Structure and avesicular mechanisms of function. J. Cell Sci..

[6] Derganc J., Mironov A.A., Svetina S. (2006). Physical factors that affect the number and size of Golgi cisternae. Traffic.

[7] Derganc J. (2007). Curvature-driven lateral segregation of membrane constituents in Golgi cisternae. Phys. Biol..

[8] Derganc J., Mironov A.A., Svetina S., Mironov A.A., Pavelka M. (2008). The geometry of organelles of the secretory pathway. The Golgi Apparatus. State of the Art 110 Years after Camillo Golgi’s Discovery.

[9] Campelo F., Kozlov M.M. (2014). Sensing membrane stresses by protein insertions. PLoS Comput. Biol..

[10] Hankins H.M., Baldridge R.D., Xu P., Graham T.R. (2015). Role of flippases, scramblases and transfer proteins in phosphatidylserine subcellular distribution. Traffic.

[11] Watanabe S., Rost B.R., Camacho-Pérez M., Davis M.W., Söhl-Kielczynski B., Rosenmund C., Jorgensen E.M. (2013). Ultrafast endocytosis at mouse hippocampal synapses. Nature.

[12] Watanabe S., Liu Q., Davis M.W., Hollopeter G., Thomas N., Jorgensen N.B., Jorgensen E.M. (2013). Ultrafast endocytosis at Caenorhabditis elegans neuromuscular junctions. Elife.

[13] Ladinsky M.S., Mastronarde D.N., McIntosh J.R., Howell K.E., Staehelin L.A. (1999). Golgi structure in three dimensions: Functional insights from the normal rat kidney cell. J. Cell Biol..

[14] Polishchuk R.S., Mironov A.A. (2004). Structural aspects of Golgi function. Cell Mol. Life Sci..

[15] Marsh B.J., Mastronarde D.N., Buttle K.F., Howell K.E., McIntosh J.R. (2001). Organellar relationships in the Golgi region of pancreatic β cell line, HIT-T15, visualized by high resolution electron tomography. Proc. Natl. Acad. Sci. USA.

[16] Beznoussenko G.V., Mironov A.A. (2002). Models of intracellular transport and evolution of the Golgi complex. Anat. Rec..

[17] Mironov A.A., Beznoussenko G.V. (2011). Molecular mechanisms responsible for formation of Golgi ribbon. Histol. Histopathol..

[18] Mironov A.A., Beznoussenko G.V. (2012). The kiss-and-run model of intra-Golgi transport. Int. J. Mol. Sci..

[19] Beznusenko G.V., Sesorova I.S., Banin V.V. (2006). Electron-tomographic analysis of the Golgi complex structure in cultured cells. Morfologiia.

[20] Mironov A.A., Sesorova I.V., Beznoussenko G.V. (2013). Golgi’s way: A long path toward the new paradigm of the intra-Golgi transport. Histochem. Cell Biol..

[21] Marsh B.J., Volkmann N., McIntosh J.R., Howell K.E. (2004). Direct continuities between cisternae at different levels of the Golgi complex in glucose-stimulated mouse islet β cells. Proc. Natl. Acad. Sci. USA.

[22] Trucco A., Polishchuk R.S., Martella O., di Pentima A., Fusella A., di Giandomenico D., San Pietro E., Beznoussenko G.V., Polishchuk E.V., Baldassarre M. (2004). Secretory traffic triggers the formation of tubular continuities across Golgi sub-compartments. Nat. Cell Biol..

[23] Bouchet-Marquis C., Starkuviene V., Grabenbauer M. (2008). Golgi apparatus studied in vitreous sections. J. Microsc..

[24] Mironov A.A., Beznoussenko G.V. (2009). Correlative microscopy: A potent tool for the study of rare or unique cellular and tissue events. J. Microsc..

[25] Mironov A.A., Mironov A.A. (1998). Estimation of subcellular organelle volume from ultrathin sections through centrioles with a discretized version of vertical rotator. J. Microsc..

[26] Mironov A.A. (1980). Structural and ionometric analysis of the mechanisms of the development of irreversible injuries in the hypothermic preservation of kidneys in a solution of the intracellular type. Arkh Patol..

[27] Murk J.L., Posthuma G., Koster A.J., Geuze H.J., Verkleij A.J., Kleijmeer M.J., Humbel B.M. (2003). Influence of aldehyde fixation on the morphology of endosomes and lysosomes: Quantitative analysis and electron tomography. J. Microsc..

[28] Kweon H.S., Beznoussenko G.V., Micaroni M., Polishchuk R.S., Trucco A., Martella O., di Giandomenico D., Marra P., Fusella A., di Pentima A. (2004). Golgi enzymes are enriched in perforated zones of golgi cisternae but are depleted in COPI vesicles. Mol. Biol. Cell..

[29] Klausner R.D., Donaldson J.G., Lippincott-Schwartz J. (1992). Brefeldin A: Insights into the control of membrane traffic and organelle structure. J. Cell Biol..

[30] Guo Q., Vasile E., Krieger M. (1994). Disruptions in Golgi structure and membrane traffic in a conditional lethal mammalian cell mutant are corrected by epsilon-COP. J. Cell Biol..

[31] Misteli T., Warren G. (1994). COP-coated vesicles are involved in the mitotic fragmentation of Golgi stacks in a cell-free system. J. Cell Biol..

[32] Fusella A., Micaroni M., di Giandomenico D., Mironov A.A., Beznoussenko G.V. (2013). Segregation of the Qb-SNAREs GS27 and GS28 into Golgi vesicles regulates intra-Golgi transport. Traffic.

[33] Yang J.S., Lee S.Y., Gao M., Bourgoin S., Randazzo P.A., Premont R.T., Hsu V.W. (2002). ARFGAP1 promotes the formation of COPI vesicles, suggesting function as a component of the coat. J. Cell Biol..

[34] Barr F.A., Puype M., Vandekerckhove J., Warren G. (1997). GRASP65, a protein involved in the stacking of Golgi cisternae. Cell.

[35] Shorter J., Watson R., Giannakou M.-E., Clarke M., Warren G., Barr F.A. (1999). GRASP55, a second mammalian GRASP protein involved in the stacking of Golgi cisternae in a cell-free system. EMBO J..

[36] Orci L., Tagaya M., Amherdt M., Perrelet A., Donaldson J.G., Lippincott-Schwartz J., Klausner R.D., Rothman J.E. (1991). Brefeldin A, a drug that blocks secretion, prevents the assembly of non-clathrin-coated buds on Golgi cisternae. Cell.

[37] Happe S., Cairns M., Roth R., Heuser J., Weidman P. (2000). Coatomer vesicles are not required for inhibition of Golgi transport by G-protein activators. Traffic.

[38] Kolpakov V., Polishchuk R., Bannykh S., Rekhter M., Solovjev P., Romanov Y., Tararak E., Antonov A., Mironov A. (1996). Atherosclerosis prone branch regions in human aorta: Microarchitecture and cell composition of intima. Atherosclerosis.

[39] Polishchuk R.S., Polishchuk E.V., Mironov A.A. (1999). Stack coalescence in MT-deprived cells with fragmented Golgi. Eur. J. Cell Biol..

[40] Mironov A.A., Colanzi A., Polishchuk R.S., Beznoussenko G.V., Mironov A.A., Fusella A., di Tullio G., Silletta M.G., Corda D., de Matteis M.A. (2004). Dicumarol, an inhibitor of ADP-ribosylation of CtBP3/BARS, fragments Golgi non-compact tubular zones and inhibits intra-Golgi transport. Eur. J. Cell Biol..

[41] Cutrona M.B., Beznoussenko G.V., Fusella A., Martella O., Moral P., Mironov A.A. (2013). Silencing of the mammalian Sar1 isoforms reveals COPII-independent protein sorting and transport. Traffic.

[42] Kreft M.E., di Giandomenico D., Beznoussenko G.V., Resnik N., Mironov A.A., Jezernik K. (2010). Golgi apparatus fragmentation as a mechanism responsible for uniform delivery of uroplakins to the apical plasma membrane of uroepithelial cells. Biol. Cell..

[43] Mironov A.A., Colanzi A., Silletta M.G., Fiucci G., Flati S., Fusella A., Polishchuk R.S., Mironov A.A., di Tillio D., Weigert R. (1997). Role of NAD+ and ADP-ribosylation in the maintenance of the Golgi structure. J. Cell Biol..

[44] Godi A., Pertile P., Meyers R., Marra P., di Tullio G., Iurisci C., Luini A., Corda D., de Matteis M.A. (1999). ARF mediates recruitment of PtdIns-4-OH kinase-β and stimulates synthesis of PtdIns(4,5)P2 on the Golgi complex. Nat. Cell Biol..

[45] Godi A., Santone I., Pertile P., Devarajan P., Stabach P.R., Morrow J.S., di Tullio G., Polishchuk R., Petrucci T.C., Luini A. (1998). ADP ribosylation factor regulates spectrin binding to the Golgi complex. Proc. Natl. Acad. Sci. USA.

[46] Vetterlein M., Ellinger A., Neumuller J., Pavelka M. (2002). Golgi apparatus and TGN during endocytosis. Histochem. Cell Biol..

[47] Evangelista V., Celardo A., Dell'Elba G., Manarini S., Mironov A., de Gaetano G., Cerletti C. (1999). Platelet contribution to leukotriene production in inflammation: *in vivo* evidence in the rabbit. Thromb Haemost..

[48] Mironov A.A., Beznoussenko G.V., Nicoziani P., Martella O., Trucco A., Kweon H.S., di Giandomenico D., Polishchuk R.S., Fusella A., Lupetti P. (2001). Small cargo proteins and large aggregates can traverse the Golgi by a common mechanism without leaving the lumen of cisternae. J. Cell Biol..

[49] Polishchuk R.S., Polishchuk E.V., Marra P., Alberti S., Buccione R., Luini A., Mironov A.A. (2000). Correlative light-electron microscopy reveals the tubular-saccular ultrastructure of carriers operating between Golgi apparatus and plasma membrane. J. Cell Biol..

[50] Russ J.C., Dehoff R.T. (2000). Practical Stereology.

[51] Orci L., Glick B.S., Rothman J.E. (1986). A new type of coated vesicular carrier that appears not to contain clathrin: Its possible role in protein transport within the Golgi stack. Cell.

[52] Barlowe C., Orci L., Yeung T., Hosobuchi M., Hamamoto S., Salama N., Rexach M.F., Ravazzola M., Amherdt M., Schekman R. (1994). COPII: A membrane coat formed by Sec proteins that drive vesicle budding from the endoplasmic reticulum. Cell.

[53] Bremser M., Nickel W., Schweikert M., Ravazzola M., Amherdt M., Hughes C.A., Söllner T.H., Rothman J.E., Wieland F.T. (1996). Coupling of coat assembly and vesicle budding to packaging of putative cargo receptors. Cell.

[54] Orci L., Schekman R., Perrelet A. (1996). Interleaflet clear space is reduced in the membrane of COP I and COP II-coated buds/vesicles. Proc. Natl. Acad. Sci. USA.

[55] Vanhecke D., Studer D., Ochs M. (2007). Stereology meets electron tomography: Towards quantitative 3D electron microscopy. J. Struct. Biol..

