# Supplementary material for: Trans-Membrane Area Asymmetry Controls the Shape of Cellular Organelles
Source: Int J Mol Sci. 2015 Mar 9;16(3):5299–333. doi: 10.3390/ijms16035299 (PMC4394477; doi:10.3390/ijms16035299)
Supplement: Supplementary file 1 [file ijms-16-05299-s001.pdf]

Supplementary Information

Controls

Although our data fully support the predictions, they are still different from the theoretical point of view. Actually, our absolute values of TAA were higher than our predictions based on the assumption that the thickness of the Golgi membranes is 4 nm. Indeed, when we used a membrane thickness of 4 nm, as has been shown by physical measurements, TAA of 52 nm COPI vesicles would be 1.4, whereas in our measurements this was 2.5. However, considering that the membrane thickness was measured after treatment with OsO_4, which leads to precipitation of Os along the internal and external surfaces of membranes, the data become reasonable. When we tested our method of TAA measurements using images published by Orci *et al*. [44], where they used tannic acid instead of thiocarbohydrazide, as in the paper by Orci *et al*. [47], the thickness of the membranes after treatment with OsO_4 and tannic acid was 10 nm. Our measurement demonstrated that in the images presented, TAA was 2.6. This demonstrates that our methods of TAA measurement are valid.

According to Marsh *et al*. [15], the surface area of the external surface of an uncoated COPI-dependent vesicle is 8495 nm^2. Our measurements revealed that when samples were prepared with the help of the protocol that included thiocarbohydrazide, the thickness of the membrane was 8 to 9 nm. Under these conditions, the surface area of the internal surface of a COPI-dependent vesicle was 3632 nm^2, whereas the ratio between the external and internal surface area was 2.34. Initially, we measured TAA in published images. An image was smoothed using ImageJ and the size of a pixel was made equal to 1 nm^2. Then, we placed the red lines on the black and white EM image, and for each situation where the upper edge of the red line moved tangentially along the membrane, this area was magnified and classified according to the scheme presented in Figure S1.

Our measurements gave unusually high levels of TAA for Golgi membranes, and especially for 52 nm vesicles. If we take into consideration that the thickness of the membrane visualized with OsO_4 is 10 nm, the disk with a diameter of 1 μm and a height of 40 nm and a toroidal rim will give the surface area of the external leaflet of 1772 nm^2, whereas the internal leaflet is 1670 nm^2, for TAA of 1.06. When COPI vesicles were tested using stereological approaches after staining with OsO_4, TAA was 2.6. The surface area of the external leaflet estimated along the very external contour was 8495 nm^2, and the internal contour gives 3215.4 nm^2. If we add ten 52-nm vesicles into a disk with a diameter of 1 μm and a height (thickness) of 40 nm, the resulting TAA of the disk will increase from 1.06 to 1.09. These values are similar to our data.

In our samples, the ratio between the external (intraluminal) surface area and the internal (inside a vesicle) surface area was 1.53. The breakdown of the microvilli into spherical vesicles gave vesicles with a diameter of 110 nm.

Table S1. Typical membrane curvature in secretory organelles. All of the values are given in terms of the typical maximal radius of curvature, R, encountered in the secretory organelles. The value of the distance between the neutral planes of the two membrane leaflets, h, is approximated by the distance between the planes of the polar lipid heads in the bilayer [8]. The total curvature is the sum of membrane curvatures in two perpendicular directions, Gaussian curvature is the product of the two curvatures, and trans-membrane area asymmetry can be calculated as: TAA = $\left(\frac{\Delta A}{A}+1\right)$, where A is the membrane surface area and ΔA is the area difference between the two membrane leaflets, $\Delta A = A_{cyt} - A_{lum}$. The area difference is related to the membrane total curvature as $\Delta A = hA\left(\frac{1}{R_1}+\frac{1}{R_2}\right)$.

**Structure**	**Total Curvature**	**Gaussian Curvature**	**TAA =** $\left(\frac{\Delta A}{A}+1\right)$	**Typical TAA** ($R = 25$ nm, $h = 4$ nm)
Vesicle (radius R)	$\frac{1}{R}+\frac{1}{R}$	$\frac{1}{R^2}$	$1+\frac{2h}{R}$	1.32
Invaginated vesicle (radius R)	$-\frac{1}{R}-\frac{1}{R}$	$\frac{1}{R^2}$	$1-\frac{2h}{R}$	0.68
Cylinder (radius R)	$\frac{1}{R}+0$	0	$1+\frac{h}{R}$	1.16
Rim of a discoid cisterna (disc radius Rd)	$\frac{1}{R}+\frac{1}{Rd}$	$\frac{1}{R}\frac{1}{Rd}$	$1+\frac{h}{R}+\frac{h}{Rd}$	~1.16
Flat cisternal side	0	0	1	1
Rim of a perforation (perforation radius Rp)	$\frac{1}{R}-\frac{1}{Rp}$	$-\frac{1}{R}\frac{1}{Rp}$	$1+\frac{h}{R}-\frac{h}{Rp}$	1 < TAA < 1.16
Neck	$\frac{1}{R}-\frac{1}{R}=0$	$-\frac{1}{R^2}$	1	1

Table S2. General relations.

**Structure**	V	A	ΔA	**TAA =** $\left(\frac{\Delta A}{A}+1\right)$
Sphere radius Rs	$Vs=\frac{4\pi}{3}Rs^3$	$As=4\pi Rs^2$	$\Delta As=4\pi dRs$	$1+\frac{d}{Rs}$
Cylinder radius Rc lenght H	$Vc=\pi Rc^2 H$	$Ac=2\pi RcH$	$\Delta Ac=\pi dH$	$1+\frac{d}{2Rc}$

Note that the spherical caps of the cylinder are neglected; (*d* is thickness of the bilayer, in the Traffic paper the equations were written in terms of *h*, which is the distance between the monolayers, *d* ~ 2 *h*).

Table S3. Fusion of two spheres with radius *Rs* = 100 nm, *d* = 4 nm.

**Structure**	**Total** V **[nm³]**	**Total** A **[nm²]**	**Total** ΔA **[nm²]**	**TAA =** $\left(\frac{\Delta A}{A}+1\right)$
One sphere, *Rs* = 100 nm,	4,188,790	125,664	5027	1.04
Two spheres, *Rs* = 100 nm	8,377,580	251,327	10,053	1.04

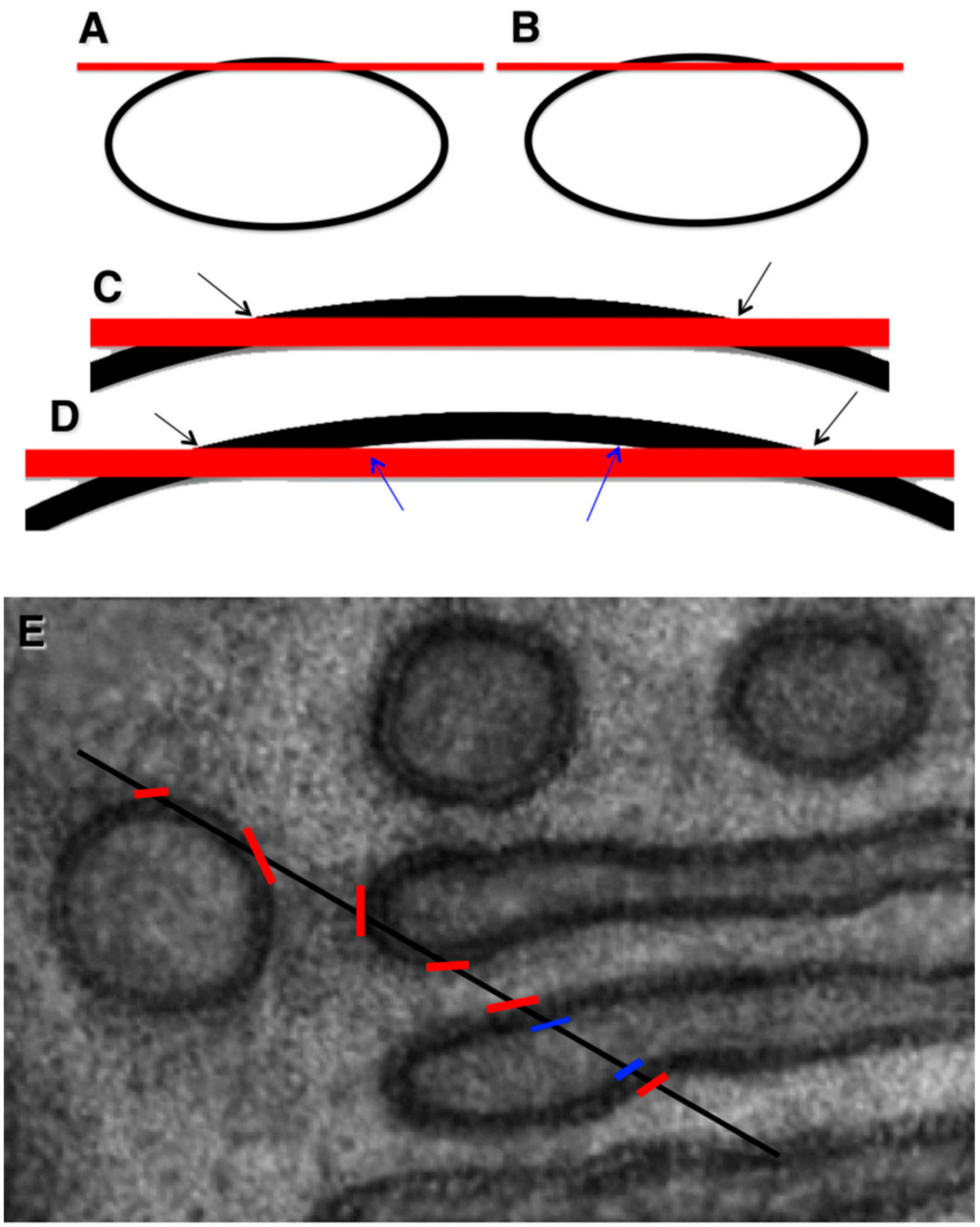

Figure S1. Estimation of membrane curvature using a stereological approach. (**A**–**D**) Scheme showing how intersections were counted. (**A**,**C**) Two intersections were counted (black arrows in **C**, as enlarged view of **A**) with the external surface of the membrane of organelle; (**B**,**D**) Two intersections were counted (**D** is enlarged area of **B**) with the external surface (black arrows), and two with the internal surface (blue arrows) of the organelle membrane; (**E**) Counting of intersections with Golgi membranes. Example of a real vesicle. Intersections between the straight black line and the red lines demonstrate the intersections between the test-line and the external contour of membranes, and intersections between the black line and the blue line demonstrate the intersections of the test-line with the luminal contours of the Golgi membranes.

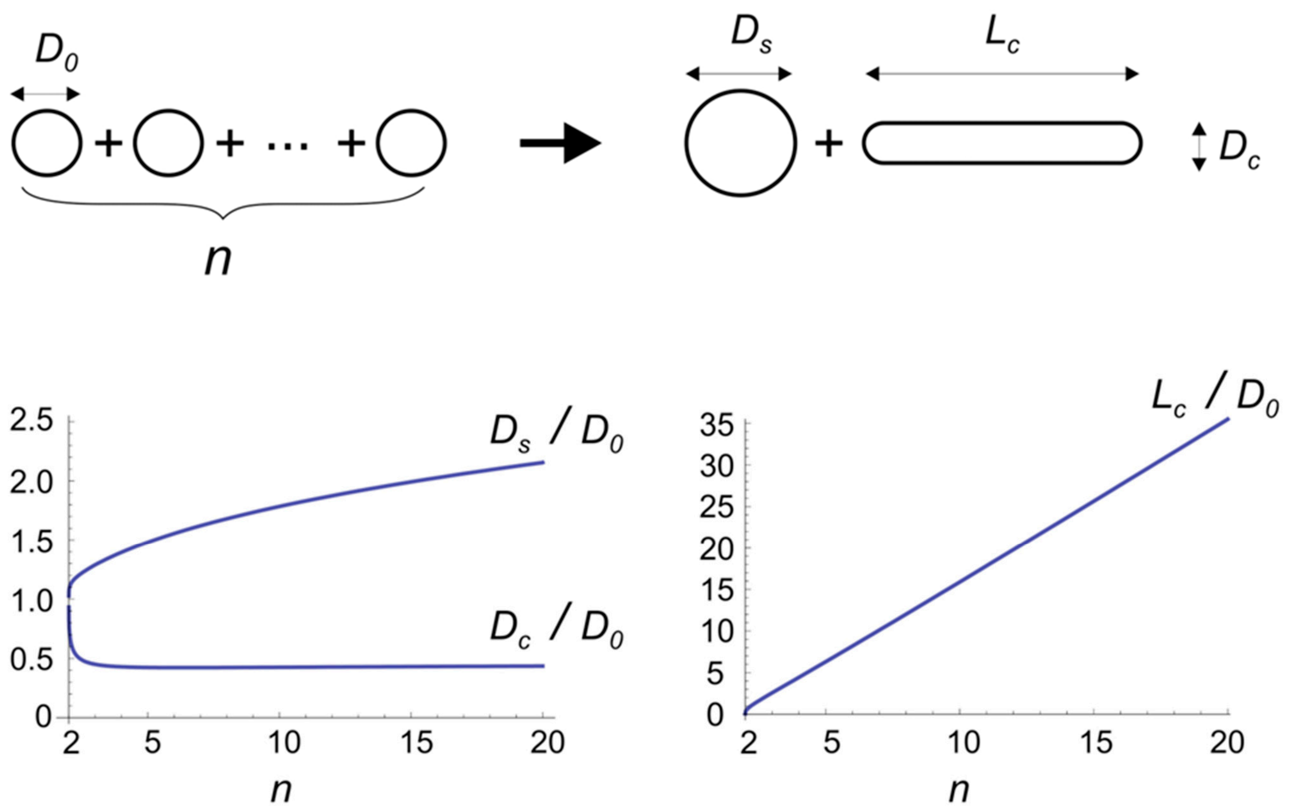

Figure S2. Scheme of fusion and results of calculations of endosomal TAA.
